# Reproducibility of Structural and Diffusion Tensor Imaging in the TACERN Multi-Center Study

**DOI:** 10.3389/fnint.2019.00024

**Published:** 2019-07-17

**Authors:** Anna K. Prohl, Benoit Scherrer, Xavier Tomas-Fernandez, Rajna Filip-Dhima, Kush Kapur, Clemente Velasco-Annis, Sean Clancy, Erin Carmody, Meghan Dean, Molly Valle, Sanjay P. Prabhu, Jurriaan M. Peters, E. Martina Bebin, Darcy A. Krueger, Hope Northrup, Joyce Y. Wu, Mustafa Sahin, Simon K. Warfield

**Affiliations:** ^1^Computational Radiology Laboratory, Department of Radiology, Boston Children’s Hospital, Harvard Medical School, Harvard University, Boston, MA, United States; ^2^Department of Neurology, Boston Children’s Hospital, Harvard Medical School, Harvard University, Boston, MA, United States; ^3^Division of Neuroradiology, Department of Radiology, Boston Children’s Hospital, Harvard Medical School, Harvard University, Boston, MA, United States; ^4^Department of Neurology, University of Alabama at Birmingham, Birmingham, AL, United States; ^5^Department of Neurology and Rehabilitation Medicine, Cincinnati Children’s Hospital Medical Center, Cincinnati, OH, United States; ^6^Department of Pediatrics, McGovern Medical School, University of Texas Health Science Center at Houston, Houston, TX, United States; ^7^Division of Pediatric Neurology, UCLA Mattel Children’s Hospital, David Geffen School of Medicine, University of California, Los Angeles, Los Angeles, CA, United States; ^8^F.M. Kirby Neurobiology Center, Boston Children’s Hospital, Harvard Medical School, Harvard University, Boston, MA, United States

**Keywords:** MRI, quality assurance, reproducibility, multicenter study, brain, ACR, phantom

## Abstract

**Background:**

Multi-site MRI studies are often necessary for recruiting sufficiently sized samples when studying rare conditions. However, they require pooling data from multiple scanners into a single data set, and therefore it is critical to evaluate the variability of quantitative MRI measures within and across scanners used in multi-site studies. The aim of this study was to evaluate the reproducibility of structural and diffusion weighted (DW) MRI measurements acquired on seven scanners at five medical centers as part of the Tuberous Sclerosis Complex Autism Center of Excellence Research Network (TACERN) multisite study.

**Methods:**

The American College of Radiology (ACR) phantom was imaged monthly to measure reproducibility of signal intensity and uniformity within and across seven 3T scanners from General Electric, Philips, and Siemens vendors. One healthy adult male volunteer was imaged repeatedly on all seven scanners under the TACERN structural and DW protocol (5 b = 0 s/mm^2^ and 30 b = 1000 s/mm^2^) over a period of 5 years (age 22–27 years). Reproducibility of inter- and intra-scanner brain segmentation volumes and diffusion tensor imaging metrics fractional anisotropy (FA) and mean diffusivity (MD) within white matter regions was quantified with coefficient of variation.

**Results:**

The American College of Radiology Phantom signal intensity and uniformity were similar across scanners and changed little over time, with a mean intra-scanner coefficient of variation of 3.6 and 1.8%, respectively. The mean inter- and intra-scanner coefficients of variation of brain structure volumes derived from T1-weighted (T1w) images of the human phantom were 3.3 and 1.1%, respectively. The mean inter- and intra-scanner coefficients of variation of FA in white matter regions were 4.5 and 2.5%, while the mean inter- and intra-scanner coefficients of variation of MD in white matter regions were 5.4 and 1.5%.

**Conclusion:**

Our results suggest that volumetric and diffusion tensor imaging (DTI) measurements are highly reproducible between and within scanners and provide typical variation amplitudes that can be used as references to interpret future findings in the TACERN network.

## Introduction

The Tuberous Sclerosis Complex Autism Center of Excellence Research Network study is a multi-center study examining neurodevelopment in infants with TSC, a rare genetic disorder associated with a high incidence (26–50%) of ASD (Jeste et al., 2008, 2014; Capal et al., 2017). One of the goals of TACERN is to acquire prospective, longitudinal structural and diffusion weighted (DW), MRI of TSC infants over the first 3 years of life, and implement advanced quantitative neuroimaging techniques to detect MRI biomarkers that predict development of ASD (Davis et al., 2017). Specifically, TACERN seeks to characterize the development of brain morphometry from structural MRI and white matter connectivity from DTI, and evaluate the relationship of these quantitative MRI measures with ASD outcome in TSC patients.

Although multi-center studies aid in recruitment of sufficiently sized samples of patients with rare conditions like TSC from diverse geographies, they also require rigorous quality control to minimize site-related bias. Multi-center, longitudinal MRI studies use multiple scanners, potentially from different vendors, and use different software to characterize deviations in quantitative MRI measures that may be associated with disease. To reliably detect disease-related changes in quantitative MRI measures, it is critical to harmonize MRI protocols across sites, adhere to strict quality control procedures, and to measure variation in MR images that may arise due to scanner-related sources of noise, and artifact (Pagani et al., 2010). Sources of variability in MR images include, but are not limited to: partial volume averaging, variations in signal intensity arising from spatially varying coil sensitivity profiles and B_1_ transmit field inhomogeneity, table vibration, thermal noise in the coils and subject that create stochastic variability in the image pixels, and geometric distortion resulting from B_0_ inhomogeneity, and gradient non-linearity (Morelli et al., 2011). The normal amplitude of hardware-induced variations in MR images can be detected and quantified using phantoms, and can be used to remove the effect of system variability from quantitative MRI measures of subjects (Keenan et al., 2018).

The American College of Radiology accreditation program has developed a designated MR protocol and phantom designed to facilitate scanner quality control. The ACR phantom is a short, hollow acrylic plastic cylinder of standard dimensions, filled with nickel chloride, and sodium chloride. Structures within the phantom allow for measurements of image quality, including SNR and image intensity uniformity (American College of Radiology, 2018). Previous reports indicate that frequent, repeat imaging of the ACR phantom is an effective method for monitoring and evaluating image quality, and is useful in multisite studies (Chen et al., 2004; Ihalainen et al., 2011; Davids et al., 2014).

However, the ACR phantom does not accurately reproduce all properties of *in-vivo* tissue, such as its microscopic diffusion properties. The lack of a validated phantom for DWI with FA and MD similar to those seen in humans makes the accurate assessment of DWI reproducibility across scanners challenging. The best alternative to date is to scan a living human phantom on each scanner. Repeated imaging of the same human on all study scanners has successfully characterized the normal physical and physiological variability in numerous multi-center studies (Vollmar et al., 2010; Fox et al., 2012; Zhu et al., 2012; Grech-Sollars et al., 2015; Palacios et al., 2017; Duchesne et al., 2019).

The goal of this work was to determine the reproducibility of MRI structural and diffusion data acquired on seven scanners over 5 years as part of the TACERN study. Monthly ACR phantom imaging was performed to measure variation in signal intensity and uniformity within and across scanners. A single healthy volunteer was also imaged on each scanner under the TACERN imaging protocol when possible with a goal of every six months at each site for a total of 26 scans. We analyzed all images using the same processing pipeline which included a fully automatic computation of the volume of brain structures and DTI parameters within 17 white matter regions. In order to assess the reproducibility, we calculated the coefficient of variation (CV) for ACR phantom intensity measures and the human phantom volumetric and DTI measures. Our results indicate good reproducibility of quantitative MRI measures across and within scanners and will inform future interpretation of MRI findings in the TACERN network.

## Materials and Methods

### Study Design and Sample

This study was performed to measure the variability of quantitative structural and DW brain MRI measurements across multiple scanners used in the TACERN study, an ongoing, prospective, longitudinal, multi-site study investigating MRI biomarkers of ASD in infants with TSC. TACERN sites include BCH, CCHMC, UAB, UCLA, and McGovern Medical School at University of Texas Health Science Center (UTH).

Image quality was evaluated with two methods: (1) The ACR phantom was imaged monthly under the standardized ACR phantom protocol to evaluate the stability of MR signal intensity and uniformity over the study period. (2) A healthy adult male volunteer was imaged under the TACERN MRI protocol on every study scanner over a period of 5 years (age 22–27 years) to evaluate the variability of quantitative MRI measurements that will be made in the TSC cohort. The human phantom was scanned every 6 months at each site, when possible. At each bi-annual scan session, scan-rescan, or back-to-back imaging of the volunteer under identical TACERN protocols with a brief exit and re-entry of the scanner between scan sessions, was achieved when possible, given the scheduling demands of the clinical scanners used in this study. Scan-rescan is valuable because it reduces the magnitude of anatomical changes that may occur with time in the subject and narrows the sources of measurement variability to those associated with the scanner and subject repositioning (Wei et al., 2004; Velasco-Annis et al., 2018). Each human phantom scan was analyzed with the fully automated TACERN MRI analysis pipeline, that includes a whole brain labeling and volumetric analysis of cortical, subcortical, cerebellar, white matter, and ventricular brain structures. The pipeline also includes a DTI analysis, which computes the single tensor field and labels regions of white matter for tract selection (pipeline described below). Brain structure volumes and white matter DTI metrics were compared across scans acquired on the same scanner (intra-scanner) and across all scanners (inter-scanner) to evaluate the reproducibility of quantitative MRI measurements. All study procedures were approved by the Institutional Review Board at each site, and the human phantom provided written informed consent.

### MRI Acquisition

MRI scans were acquired at 3T on seven scanners and five scanner models, including one GE Signa HDxt, one Philips Achieva, two Philips Ingenia, one Siemens Skyra and two Siemens TrioTim scanners with 32, 12, and 8 channel head coils. Software upgrades occurred on two of the seven scanners during the course of the study (Table 1). Scanner B replaced scanner A at BCH after 3.7 years of research use and scanner E replaced scanner D at CCHMC after 1.5 years of research use.

**TABLE 1:** Clinical T1, T2, and Diffusion-weighted MR protocols for the TACERN study.

	**BCH**		**UCLA**	**CCHMC**		**UAB**	**UTH**
ScannerID	A	B	C	D	E	F	G
Field strength (T)	3	3	3	3	3	3	3
Manufacturer	Siemens	Siemens	Siemens	Philips	Philips	Philips	General Electric
Model	TrioTim	Skyra	TrioTim	Achieva	Ingenia	Ingenia	Signa HDxt
Software versions	syngoMRB17	syngoMRE11	syngoMRB17	3.2.1	5.1.9; 5.3.0	4.1.3; 5.1.7; and 5.3.0	HD 16
Number of head coil channels	32	32	12	32	32	32	8

T1-weighted							
Orientation	sagittal	sagittal	sagittal	sagittal	sagittal	sagittal	sagittal
Field of view (mm)	256 × 256	224 × 224	256 × 256	220 × 220	220 × 220	220 × 220	220 × 220
Matrix	256 × 256	256 × 256	256 × 256	224 × 224	224 × 224	224 × 224	256 × 256
Number of slices	176	192	176	176	176	176	172
Resolution (mm)	1.0 × 1.0 × 1.0	0.9 × 0.9 × 0.9	1.0 × 1.0 × 1.0	1.0 × 1.0 × 1.0	0.9 × 0.9 × 1.0	1.0 × 1.0 × 1.0	0.9 × 0.9 × 1.0
Repetition time (ms)	8	8	8	8	8	8	6
Echo time (ms)	4	2	4	4	4	4	3
Bandwidth (Hz/Px)	199	200	199	191	191	191	244
Inversion time (ms)	1100	1100	1100	1100	1100	1100	1100
Flip angle (deg)	7	7	7	7	7	7	7
Number of averages	1	1	1	1	1	1	1

T2-weighted							
Orientation	axial	axial	axial	axial	axial	axial	axial
Field of view (mm)	159 × 200	162 × 200	159 × 200	200 × 200	200 × 200	200 × 200	200 × 200
Matrix	408 × 512	364 × 448	408 × 512	512 × 512	512 × 512	512 × 512	512 × 512
Number of slices	76	90	76	76	76	76	76
Resolution (mm)	0.4 × 0.4 × 2.0	0.4 × 0.4 × 2.0	0.4 × 0.4 × 2.0	0.4 × 0.4 × 2.0	0.4 × 0.4 × 2.0	0.4 × 0.4 × 2.0	0.4 x 0.4 × 2.0
Repetition time (ms)	14850	10900	14850	9366	7182	10300	15000
Echo time (ms)	79	82	79	79	79	79	76
Bandwidth (Hz/Px)	208	225	208	196	200	196	244
Flip angle (deg)	90	90	90	90	90	90	90
Number of averages	2	2	2	2	2	2	2

Diffusion-weighted							
Orientation	axial	axial	axial	axial	axial	axial	axial
Field of view (mm)	220 × 220	220 × 220	220 × 220	220 × 220	220 × 220	220 × 220	220 × 220
Matrix	128 × 128	128 × 128	128 × 128	128 × 128	128 × 128	128 × 128	256 × 256
Number of slices	74	74	74	68	72	72	48
Resolution (mm)	1.7 × 1.7 × 2.0	1.7 × 1.7 × 2.0	1.7 × 1.7 × 2.0	1.7 × 1.7 × 2.0	1.7 × 1.7 × 2.0	1.7 × 1.7 × 2.0	0.9 × 0.9 × 2.0
Repetition time (ms)	6448	6800	10900	10400	11300	15000	12700
Echo time (ms)	88	94	88	64	98	78	87
Bandwidth (Hz/Px)	1395	1500	1395	2378	1144	1276	1953
Flip Angle (deg)	90	90	90	90	90	90	90
Number of averages	1	1	1	1	1	1	1
*b*-values (number of directions)	0 (13)	0 (13)	0 (15)	0 (3)	0 (18)	0 (24)	0 (15)
	400 (6)	400 (6)	–	–	400 (6)	–	–
	600 (6)	600 (6)	–	–	600 (6)	–	–
	800 (6)	800 (6)	–	–	800 (6)	–	–
	1000 (30)	1000 (30)	1000 (30)	1000 (30)	1000 (30)	1000 (30)	1000 (30)
	1050–1850 (20)	1050–1850 (20)	1050–1850 (20)	1050–1850 (20)	1050–1850 (20)	1050–1850 (20)	–
	2000 (6)	2000 (6)	2000 (6)	2000 (6)	2000 (6)	2000 (6)	–
	–	–	2500 (30)	2500 (30)	2500 (30)	2500 (30)	2500 (30)
	3000 (4)	3000 (4)	3000 (4)	3000 (4)	3000 (4)	3000 (4)	3000 (31)

Monthly ACR Phantom scans were acquired on all study scanners under the standardized ACR phantom MRI protocol, which includes an axial T1w fast spin echo (matrix = 256 × 256, FOV = 250 mm, number of slices = 11, slice thickness = 5.0 mm, slice gap = 5.0 mm, resolution = 1.0 mm^3^ × 1.0 mm^3^ × 10.0 mm^3^, TR = 500 ms, TE = 20 ms, and Flip angle = 90 deg) and axial T2w fast spin echo (geometry matched to ACR T1w, TR = 2000 ms, TE = 20, and 80 ms).

Human phantom scans were performed awake or in natural sleep under the TACERN consensus clinical imaging protocol that includes high resolution, routine clinical imaging sequences used for annual surveillance imaging of TSC patients, plus additional multi *b*-value DW research sequences. Imaging protocols were harmonized to the extent permitted by each platform. Acquisition parameters used on each scanner are detailed in Table 1. The protocol includes a 1.0 mm^3^ × 1.0 mm^3^ × 1.0 mm^3^ sagittal T1w image, 0.4 mm^2^ × 0.4 mm^2^ in-plane resolution axial T2w image, 30 high angular resolution b = 1000 s/mm^2^, and 6 b = 0 s/mm^2^ DW images at 1.7 mm^2^ × 1.7 mm^2^ in-plane resolution and 2.0 mm slice thickness. One b = 0 s/mm^2^ DWI was acquired with reversed phase-encoding direction for distortion compensation, covering the entire brain.

### Quality Assurance

MRI data were transmitted to and evaluated at the Computational Radiology Lab at BCH. MRI metadata were reviewed for protocol compliance. Scans that did not adhere to study protocols were excluded (15 ACR, 0 human phantom). Images were reviewed by an expert rater for extent of brain coverage and artifacts resulting from a variety of sources, including but not limited to subject motion, flow, radiofrequency leak, table vibration, magnetic susceptibility, and venetian blind artifact. Artifacts were not found in ACR T1w images or human phantom T1w, T2w, or DW images.

### ACR MRI Processing

**FIGURE 1:**
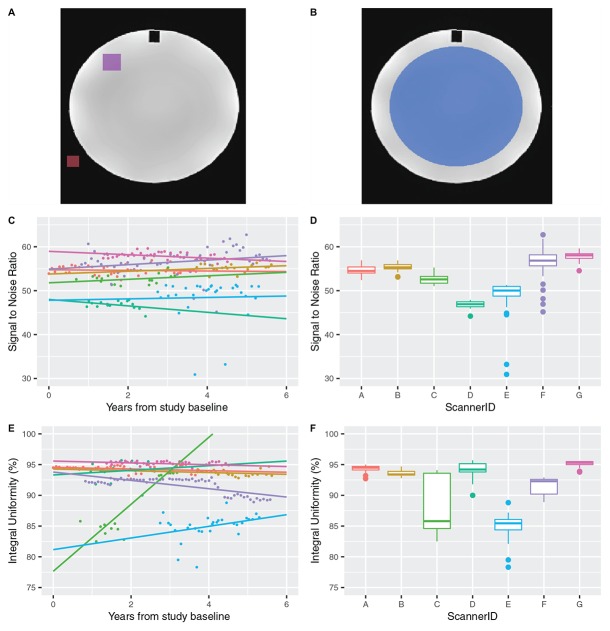
**(A)** A signal ROI (purple) and a background ROI (red) are used to calculate the SNR in the ACR phantom T1w image. **(B)** A large, circular ROI (blue) overlaid on an ideally uniform region of the ACR phantom T1w image is used to measure percent IU. **(C)** Plot of SNR over time and **(D)** by scanner for ACR phantom T1w image. **(E)** Plot of percent IU over time and **(F)** by scanner for ACR phantom T1w image.

All MRI processing and analyses were completed using the Computational Radiology Kit^1^. ACR phantom processing was completed using a fully automated processing pipeline. Each ACR phantom T1w image was aligned to a common reference ACR T1w image using rigid registration with mutual information metric. Regions of interest (ROI) were drawn on the common ACR T1w reference, as defined by the ACR Phantom Guide, and were used to measure SNR and IU (Figure 1; American College of Radiology, 2018).

A signal ROI was drawn on axial slices 6 through 10 in a uniform, high signal region of the template ACR phantom (volume = 21028 mm^3^, area/slice = 400 mm^2^). A background ROI was drawn on axial slices 2 through 10 (volume = 18024 mm^3^, area/slice = 182 mm^2^) in the background adjacent to the ACR phantom. The SNR was calculated using the mean of the signal ROI, ${\bar{x}}_{S\,i\,g\,n\,a\,l}$, and the SD of the background ROI, σ_*Background*_, as follows:

$$S\,N\,R=\frac{{\bar{x}}_{S\,i\,g\,n\,a\,l}}{\sigma_{B\,a\,c\,k\,g\,r\,o\,u\,n\,d}}$$

Integral uniformity was measured in a large, circular uniform region on slice 7 of the template ACR phantom (volume = 1746687 mm^3^; area = 174669 cm^2^) (Figure 1). Voxels within the ROI were ordered from low to high intensity, and the image intensities of the 5th (low) and 95th (high) percentile voxels were identified and used to calculate IU as described in (Fu et al., 2006):

$$I\,U=100\times \left(1-\left[\frac{h\,i\,g\,h-l\,o\,w}{h\,i\,g\,h+l\,o\,w}\right]\right)$$

### Human Phantom Structural MRI Processing

All MRI processing and analyses were completed using the Computational Radiology Kit (see text footnote 1). Human phantom processing was completed using a fully automated processing pipeline. In the native space of each human phantom scan, the T2w image was aligned and resampled to the 1.0 mm^3^× 1.0 mm^3^ × 1.0 mm^3^ T1w image using rigid registration with mutual information metric. The ICC was then segmented using a previously validated multispectral ICC segmentation method (Grau et al., 2004), and the ICC was masked from the T1w and T2w images.

Next, a fully automatic, multi-template MRI parcellation approach was used to parcellate the T1w image into ROI for volumetric analysis. We constructed a template library, composed of 18 T1w images of healthy controls, each with manual cortical, subcortical, white matter, cerebellar, and ventricular segmentations based on well-established MRI brain labeling protocols provided by the Center for Morphometric Analysis at Massachusetts General Hospital^2^ (Caviness et al., 1996; Klein and Tourville, 2012). The 18 templates were each non-linearly aligned to each subject using dense registration between the T1w anatomical scans. The dense deformation field was then used to resample the template manual segmentations to the target subject anatomy, resulting in 18 template segmentations aligned to the target T1w image. A consensus segmentation was computed from all aligned segmentations using the PSTAPLE algorithm (Akhondi-Asl and Warfield, 2013). PSTAPLE uses both the label images and intensity profiles of the T1w templates to compute probability maps for each target structure, ultimately leading to a fully automatic consensus labeling of each brain. Finally, the volume of each label (*n* = 38) was computed. Subcortical and cortical volume measurements estimated by PSTAPLE have been shown to be more reproducible and accurate than Freesurfer and other similar algorithms (Velasco-Annis et al., 2018).

### Human Phantom DW MRI Processing

The DW images were corrected for magnetic susceptibility distortion using the pair of b = 0 s/mm^2^ images with opposite phase-encoding direction and FSL top-up (Andersson et al., 2003). Inter-volume motion correction was then performed by affine registration of each DW image to the average b = 0 s/mm^2^ image. The DW images were aligned and up-sampled to the 1.0 mm^3^ × 1.0 mm^3^ × 1.0 mm^3^ T2w resampled scan using affine registration and sinc interpolation, and the brain extracted on DWI using the previously computed ICC segmentation (Dyrby et al., 2014). A single tensor diffusion model was estimated using robust least squares in each brain voxel from which fractional anisotropy [FA = 3Var(λ)/(λ^2^_1_ + λ^2^_2_ + λ^2^_3_)^1/2^] and mean diffusivity [MD = (λ_1_ + λ_2_ + λ_3_)/3] were computed, where λ_*i*_ represent the eigenvalues of the diffusion tensor (Mori and Zhang, 2006).

Next, a fully automatic, multi-template approach was used to define 17 white matter ROIs in the native space of each human phantom DTI scan using a previously validated method (Suarez et al., 2012). A template library was constructed from whole brain DTI of 20 healthy controls, with each scan in its native space. The DTI were computed from 30 high angular resolution b = 1000 s/mm^2^ and 5 b = 0 s/mm^2^ TACERN protocol DW images.

For each template, scalar FA and color maps of the principal diffusion directions were computed from the DTI. ROI were hand drawn by an expert rater on the color map within white matter fiber bundles following previously defined and validated labeling schemes for tractography (Catani et al., 2005; Catani and Thiebaut de Schotten, 2008; Benjamin et al., 2014). To delineate the same white matter ROIs in the native space of each human phantom scan, the following procedure was performed for every template: the template scalar FA map was aligned to the target human phantom scalar FA map using affine registration with mutual information metric. The affine registration field was used to initialize a non-linear, dense registration of the template DTI to the human phantom DTI. The affine and dense deformation fields were then used to resample the template white matter ROIs to the human phantom native DTI space using nearest neighbor interpolation. Now with 20 sets of white matter ROIs (one for each template) aligned to the native space of the human phantom scan, a final, consensus set of white matter ROIs was computed using the STAPLE algorithm (Warfield et al., 2004). Lastly, mean FA and MD were computed in each ROI.

### White Matter ROIs

The ROIs analyzed in this analysis were defined using previously validated labeling schemes for tractography and include left and right posterior limb of the internal capsule, anterior limb of the internal capsule, cingulum body, corpus callosum, and inferior extreme capsule, from here on referred to as uncinate fasciculus (Catani and Thiebaut de Schotten, 2008). The sagittal stratum was defined following the labeling technique for tractography of the optic radiations presented in (Benjamin et al., 2014). Three ROIs were placed along the arcuate fasciculi in each hemisphere; in the white matter (1) projecting from the inferior parietal lobule to the inferior frontal gyrus, (2) underlying the inferior parietal lobule, and (3) underlying the posterior superior temporal gyrus, following the labeling scheme presented in (Catani et al., 2005). From here on we refer to these ROIs as left and right arcuate fasciculus region 1, region 2, and region 3, respectively.

### Statistical Analysis

We quantified reproducibility using the coefficient of variation (CV) of quantitative MR measurements. The inter-scanner (all scans across all scanners) and intra-scanner (all scans across a single scanner) CV were measured for SNR and IU of the ACR phantom, brain structure volume measurements derived from brain segmentation labels, and for FA and MD of white matter, measured within white matter labels. Intra-vendor (all scans across a single scanner vendor) CV was also computed. The CV of an MR measurement is defined as the ratio of the SD (σ) to the mean ($\bar{x})$ of the measurement, expressed as a percentage:

$$\text{Inter-scanner CV:}\;C\,V_{j}=\frac{\sigma_{j}}{{\bar{x}}_{j}}\times 100\%$$

$$\text{Intra-scanner CV:}\;C\,V_{i\,j}=\frac{\sigma_{i\,j}}{{\bar{x}}_{i\,j}}\times 100\%$$

$$\text{Intra-vendor CV:}\;C\,V_{k}=\frac{\sigma_{k\,j}}{{\bar{x}}_{k\,j}}\times 100\%$$

where *i* indexes scanner, *j* indexes label, and *k* indexes scanner vendor.

A CV of value 0 would represent perfect reproducibility, while a greater value represents a larger SD relative to the mean of the sample. CV is an ideal measure of reproducibility of brain volume measurements because it is a dimensionless value relative to the size of the structure of interest. The analysis was completed using R software version 3.5.1.

## Results

### ACR Phantom

There were 216 ACR phantom scans in total acquired on 7 of 7 TACERN scanners available for analysis (Table 2). Results of SNR and IU variability over the study period are presented in Figure 1 and Table 3. SNR was highest on scanner G at 57 ± 1 and lowest on scanner D at 46.8 ± 0.9. SNR was most variable on scanner E, with a CV of 9.9%. Overall, SNR variability was low over the study period, with CV less than 2.1% on 5 of 7 scanners evaluated.

**TABLE 2:** Scan Information.

	**Scanner**	**A**	**B**	**C**	**D**	**E**	**F**	**G**	**Overall**
ACR	Number scans (% of total)	38 (18)	21 (10)	17 (8)	15 (7)	30 (13)	57 (26)	38 (18)	216
	Years over which scans were acquired	3.7	1.9	2.5	1.5	2.5	4.8	3.1	
Human phantom	Number scans (% of total)	4 (15)	2 (8)	2 (8)	3 (12)	4 (15)	7 (27)	4 (15)	26
	Number re-scans	2	1	0	1	2	3	0	9
	Years over which scans were acquired	0.8	0	3.0	0.9	1.4	4.5	4.7	

*ACR, American College of Radiology.*

**TABLE 3:** Variability of ACR Phantom T1-weighted signal to noise ratio and percent integral uniformity over the study period.

	**Signal to noise ratio**			**Integral uniformity (%)**		
**Scanner**	**Mean**	**SD**	**CV (%)**	**Mean (%)**	**SD (%)**	**CV (%)**
A	54	1	1.8	95.1	0.5	0.6
B	55.3	0.9	1.7	94.3	0.6	0.5
C	52.6	1.1	2.1	88.8	4.9	5.5
D	46.8	0.9	2.0	91.7	1.3	1.7
E	48.5	4.8	9.9	94.2	0.5	2.4
F	56.5	3.3	5.8	93.6	0.5	1.4
G	57	1	1.7	85.0	2.1	0.5

Average IU was highest on scanner A at 95.1% and lowest on scanner G at 85.0%. IU was most variable on scanner C, with a CV of 5.5%. Overall, IU was high for all scanners and IU variability was low, with an overall mean IU of 91.8% and a CV less than 2.4% on 6 of 7 scanners evaluated.

### Human Phantom Volumetric Analysis

There were 26 human phantom scans acquired on 7 of 7 TACERN scanners available for analysis. Scan and re-scan following exit and re-entry to the scanner was possible on 5 of 7 scanners in 9 of 17 scan sessions (Table 2).

**FIGURE 2:**
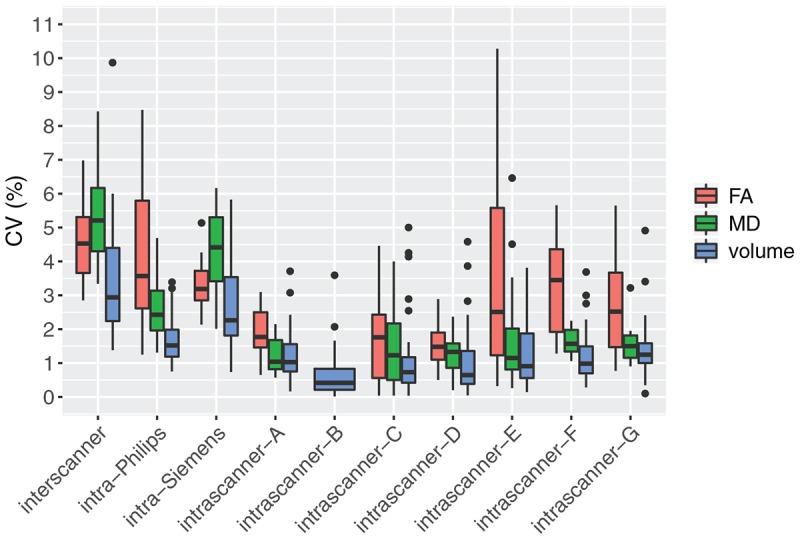
Average inter-scanner, intra-scanner, and intra-vendor variability of all brain parcellation cortical label volumes, all white matter ROI FA, and all white matter ROI MD. Intra-GE was not computed because only one GE scanner was used in the study. DTI scans were not available from scanner B.

Figure 2 and Table 4 display a summary of average inter- and intra-scanner volume CV across all labels. The average inter-scanner volume CV across all labels was 3.3%, and the average intra-scanner volume CV was 1.1% across all labels. Scanner B was the least variable scanner overall, with an average CV of 0.7% across all labels. Scanner G was the most variable scanner overall with an average CV of 1.4% across all labels. Intra-vendor CVs were also computed. The mean CV across all labels in Philips scans only was 1.7%, while the mean CV across all labels in Siemens scans was more variable, at 2.7%. There is a single GE scanner used in the study, and thus intra-vendor CV was not computed for GE.

**TABLE 4:** Average inter-scanner, intra-scanner, and intra-vendor variability of volume, FA, and MD in all labels.

	**Volume**		**FA**		**MD**	
	**Mean CV (%)**	**SD CV (%)**	**Mean CV (%)**	**SD CV (%)**	**Mean CV (%)**	**SD CV (%)**
Inter-scanner	3.3	1.6	4.5	1.2	5.4	1.4
Mean intra-scanner	1.1	0.2	2.5	0.9	1.5	0.2
Intra-scanner-A	1.3	0.8	1.9	0.7	1.2	0.5
Intra-scanner-B	0.7	0.7	–	–	–	–
Intra-scanner-C	1.1	1.2	1.7	1.2	1.5	1.2
Intra-scanner-D	1.0	1.0	1.6	0.7	1.3	0.5
Intra-scanner-E	1.2	1.0	3.7	3.2	1.8	1.7
Intra-scanner-F	1.2	0.8	3.3	1.4	1.7	0.4
Intra-scanner-G	1.4	0.9	2.7	1.4	1.5	0.5
Intra-vendor-Philips	1.7	0.7	4.0	2.0	2.6	0.9
Intra-vendor-Siemens	2.7	1.3	3.3	0.7	4.4	1.2

*CV, coefficient of variation; FA, fractional anisotropy; MD, mean diffusivity (mm^2^/s). Intra-General Electric not computed because only one General Electric scanner.*

**FIGURE 3:**
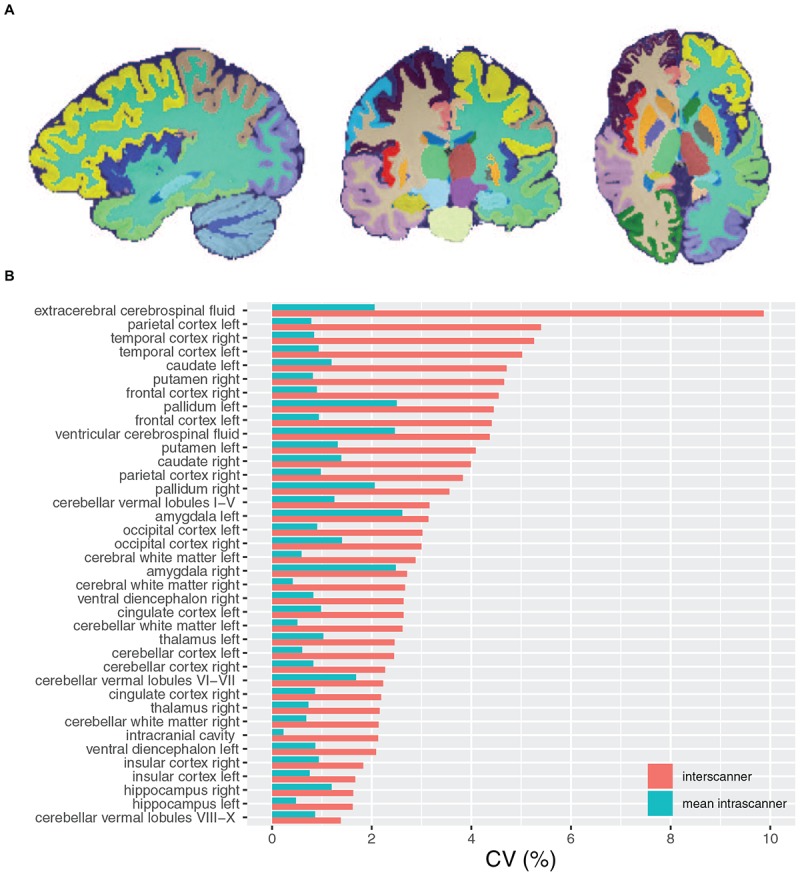
**(A)** Sagittal, coronal, and axial views of a fully automatic brain parcellation result. Each color label identifies a brain structure of interest. **(B)** Inter-scanner and mean intra-scanner CV of brain parcellation label volumes.

Figure 3 and Table 5 display the inter-scanner and mean intra-scanner mean, SD and CV of volume for each label. For purposes of concision, mean, SD, and CV for each label on each scanner are presented in Supplementary Figure 1 and Supplementary Table 1. All inter-scanner label CVs were less than 5% with the exception of right temporal cortex (5.3%), left parietal cortex (5.4%), and extracerebral spinal fluid (9.9%). The least variable label volume across scanners was the cerebellar vermis, in the region of lobules 8, 9, and 10 (1.4%). Inter-scanner CV of left and right hippocampi and insular cortex were also less than 2%.

**TABLE 5:** Inter and mean intra-scanner variability of brain parcellation label volumes.

**Label**	**Measure**	**Mean**	**SD**	**CV (%)**	**Inter: intra CV ratio**	**Mean**	**SD**	**CV (%)**	**Inter: intra CV ratio**
		**LEFT**				**RIGHT**			
Cerebellar cortex	inter-scanner	51274	1258	2.5	3.1	51490	1171	2.3	2.3
	Mean intra-scanner	51270	428	0.8		51501	521	1.0	
Cingulate cortex	Inter-scanner	12266	324	2.6	2.2	10869	238	2.2	2.2
	Mean intra-scanner	12338	147	1.2		10919	112	1.0	
Frontal cortex	Inter-scanner	94435	4163	4.4	3.1	96357	4384	4.6	3.3
	Mean intra-scanner	94040	1306	1.4		95936	1307	1.4	
Insular cortex	Inter-scanner	6356	106	1.7	1.9	6773	124	1.8	1.6
	Mean intra-scanner	6376	56	0.9		6804	72	1.1	
Occipital cortex	Inter-scanner	33598	1015	3.0	2.5	36392	1090	3.0	1.9
	Mean intra-scanner	33594	394	1.2		36295	584	1.6	
Parietal cortex	Inter-scanner	48671	2627	5.4	3.9	47279	1812	3.8	2.9
	Mean intra-scanner	48449	657	1.4		47204	631	1.3	
Temporal cortex	Inter-scanner	61785	3102	5.0	3.3	61533	3238	5.3	3.8
	Mean intra-scanner	61524	893	1.5		61207	853	1.4	
Cerebellar white matter	Inter-scanner	18600	487	2.6	3.3	18438	394	2.1	2.33
	Mean intra-scanner	18653	144	0.8		18491	160	0.9	
Cerebral white matter	Inter-scanner	245773	7068	2.9	3.2	249787	6668	2.7	3.9
	Mean intra-scanner	246668	2161	0.9		250626	1745	0.7	
Amygdala	Inter-scanner	1307	41	3.1	1.1	1289	35	2.7	1.1
	Mean intra-scanner	1309	35	2.7		1287	32	2.5	
Caudate	Inter-scanner	4130	195	4.7	2.9	4270	170	4.0	2.4
	Mean intra-scanner	4164	67	1.6		4305	73	1.7	
Hippocampus	Inter-scanner	4038	65	1.6	2.7	3984	65	1.6	1.2
	Mean intra-scanner	4053	25	0.6		3998	50	1.3	
Pallidum	Inter-scanner	1610	72	4.5	1.6	1666	59	3.5	1.5
	Mean intra-scanner	1622	44	2.8		1672	37	2.3	
Putamen	Inter-scanner	5432	222	4.1	2.4	5274	246	4.7	3.6
	Mean intra-scanner	5442	91	1.7		5299	68	1.3	
Thalamus	Inter-scanner	7903	194	2.5	2.1	7479	161	2.2	2.4
	Mean intra-scanner	7942	95	1.2		7517	68	0.9	
Ventral diencephalon	Inter-scanner	5592	117	2.1	2.1	5538	146	2.6	2.4
	Mean intra-scanner	5618	57	1.0		5564	58	1.1	

**BILATERAL**									
Cerebellar vermal lobules I-V	Inter-scanner	4699	148	3.2	2.1				
	Mean intra-scanner	4715	70	1.5					
Cerebellar vermal lobules VI-VII	Inter-scanner	1530	34	2.2	1.2				
	Mean intra-scanner	1529	27	1.8					
Cerebellar vermal lobules VIII-X	Inter-scanner	2938	40	1.4	1.6				
	Mean intra-scanner	2935	27	0.9					
Extracerebral cerebrospinal fluid	Inter-scanner	262844	25930	9.9	3.3				
	Mean intra-scanner	267104	8091	3.0					
Intracranial cavity	Inter-scanner	1553414	33165	2.1	4.2				
	Mean intra-scanner	1560739	7326	0.5					
Ventricular cerebrospinal fluid	Inter-scanner	19614	858	4.4	1.6				
	Mean intra-scanner	19758	527	2.7					

*All scans were included *(n* = 26).*

The mean intra-scanner label CV across all labels was 1.1% and within-label ranged from 0.5 to 3.0% for the ICC and extracerebral spinal fluid volumes, respectively (Tables 4, 5). The inter-scanner CV exceeded the mean intra-scanner CV by a factor of 2.5 on average and ranged from a factor of 1.1 in the right amygdala to a factor of 4.2 in the ICC.

### Human Phantom DTI ROI Analysis

There were 24 human phantom scans acquired with DWI on 6 of 7 TACERN scanners available for analysis. DTI data were not available for scanner B. Scan and re-scan following exit and re-entry to the scanner was possible on 4 of 6 scanners in 8 of 16 scan sessions (Table 2).

Figure 2 and Table 4 display a summary of inter- and intra-scanner FA and MD CV across all white matter labels. Overall, FA and MD in white matter labels were more variable within and across scanners than volume of brain segmentation labels. The average inter-scanner FA and MD CV across all labels was 4.5 and 5.4%, respectively. The average intra-scanner FA and MD CV across all labels was 2.5 and 1.5%, respectively. Scanners A and D were the least variable scanner overall, with average FA CVs of 1.9 and 1.6% and average MD CVs of 1.2 and 1.3%, respectively. Scanner E was the most variable scanner overall with an average FA CV of 3.7 % and an average MD CV of 1.8%. The mean FA CV across all labels in Philips scans slightly exceeded that of Siemens scans; with a mean Philips FA CV of 4.0% and a mean Siemens FA CV of 3.3%. In contrast, the mean MD CV across all labels in all Philips scans was lower than Siemens, with a mean Philips MD CV of 2.6%, compared to a mean Siemens MD CV of 4.4%. There is a single GE scanner used in the study, and thus intra-vendor CV was not computed for GE.

**FIGURE 4:**
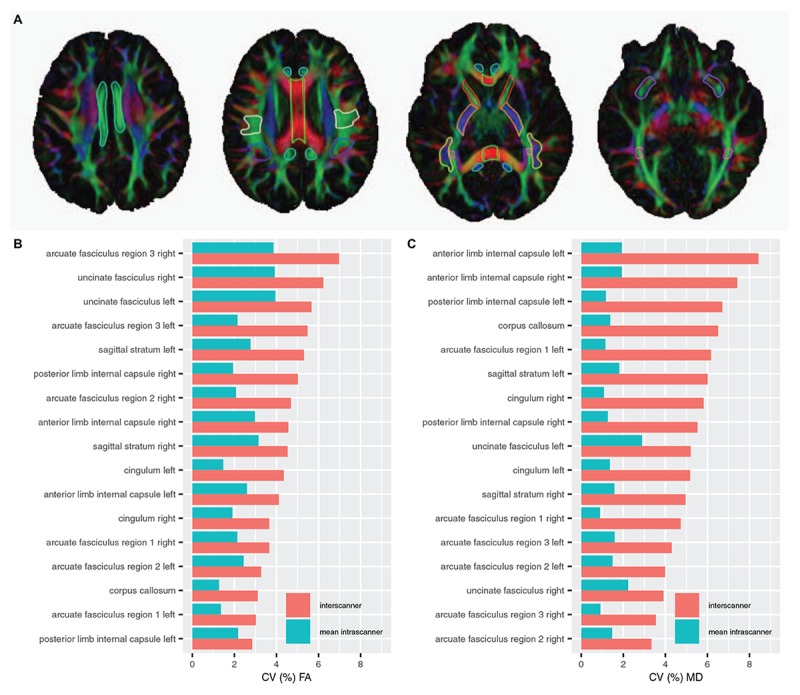
**(A)** White matter ROI superimposed on a color map of the principal diffusion directions. Red color map voxels indicate left-right diffusion, green color map voxels indicate anterior-posterior diffusion, blue color map voxels indicate inferior-superior diffusion, and other colors indicate intermediate diffusion directions. Four axial slices from a single scan depict 2D slices of 3D white matter ROI, outlined in unique colors: light blue, cingulum; green, corpus callosum; white, arcuate fasciculus region 1; royal blue, arcuate fasciculus region 2; red, anterior limb of the internal capsule; orange, posterior limb of the internal capsule; yellow, arcuate fasciculus region 3; pink, sagittal stratum; and purple, uncinate fasciculus. **(B)** Inter-scanner and mean intra-scanner CV of white matter ROI FA. **(C)** Inter-scanner and mean intra-scanner CV of white matter ROI MD. Labels are ordered from bottom to top by increasing inter-scanner coefficient of variation.

Figure 4 and Tables 6, 7 display the mean, SD and inter and intra-scanner CV of FA and MD in all white matter labels. For purposes of concision, mean, SD, and CV of FA and MD for each label on each scanner are presented in Supplementary Figure 1 and Supplementary Tables 2, 3.

**TABLE 6:** Inter and mean intra-scanner variability of FA in white matter ROIs.

**Label**	**Measure**	**Mean FA**	**SD FA**	**CV (%) FA**	**Inter: intra CV ratio**	**Mean FA**	**SD FA**	**CV (%) FA**	**Inter: Intra CV ratio**
		**LEFT**				**RIGHT**			
Anterior limb internal capsule	Inter-scanner	5.1	0.2	3.9	2.0	5.2	0.2	3.8	1.0
	Mean intra-scanner	5.1	0.1	2.0		5.2	0.2	3.8	
Arcuate fasciculus region 1	Inter-scanner	4.1	0.1	2.4	1.0	4.3	0.2	4.7	2.0
	Mean intra-scanner	4.1	0.1	2.4		4.3	0.1	2.3	
Arcuate fasciculus region 2	Inter-scanner	3.8	0.1	2.6	1.0	4.2	0.2	4.8	2.0
	Mean intra-scanner	3.8	0.1	2.6		4.2	0.1	2.4	
Arcuate fasciculus region 3	Inter-scanner	4.6	0.3	6.5	3.0	4.0	0.3	7.5	1.5
	Mean intra-scanner	4.6	0.1	2.2		4.0	0.2	5.0	
Cingulum	Inter-scanner	4.6	0.2	4.4	2.0	4.5	0.2	4.4	2.0
	Mean intra-scanner	4.6	0.1	2.2		4.5	0.1	2.2	
Posterior limb internal capsule	Inter-scanner	5.8	0.2	3.4	2.0	5.9	0.3	5.1	3.0
	Mean intra-scanner	5.8	0.1	1.7		5.8	0.1	1.7	
Sagittal stratum	Inter-scanner	5.0	0.3	6.0	3.0	4.6	0.2	4.4	2.0
	Mean intra-scanner	5.0	0.1	2.0		4.6	0.1	2.2	
Uncinate fasciculus	Inter-scanner	4.1	0.2	4.9	1.0	3.8	0.2	5.3	1.0
	Mean intra-scanner	4.2	0.2	4.8		3.8	0.2	5.3	

**BILATERAL**									
Corpus callosum	Inter-scanner	6.1	0.2	3.3	1.9				
	Mean intra-scanner	6.0	0.1	1.7					

*All scans were included (*n* = 24). DTI data were not available for Scanner B. FA is scaled × 10.*

**TABLE 7:** Inter and intra-scanner variability of MD in white matter ROIs.

**Label**	**Measure**	**Mean MD**	**SD MD**	**CV (%) MD**	**Inter: intra CV ratio**	**Mean MD**	**SD MD**	**CV (%) MD**	**Inter: Intra CV ratio**
		**LEFT**				**RIGHT**			
Anterior limb internal capsule	Inter-scanner	7.3	0.6	8.2	5.9	7.4	0.6	8.1	5.8
	Mean intra-scanner	7.2	0.1	1.4		7.3	0.1	1.4	
Arcuate fasciculus region 1	Inter-scanner	7.2	0.4	5.6	4.0	7.3	0.4	5.5	3.9
	Mean intra-scanner	7.2	0.1	1.4		7.3	0.1	1.4	
Arcuate fasciculus region 2	Inter-scanner	7.5	0.3	4.0	3.1	7.3	0.2	2.7	1.9
	Mean intra-scanner	7.5	0.1	1.3		7.3	0.1	1.4	
Arcuate fasciculus region 3	Inter-scanner	7.5	0.3	4.0	3.1	7.6	0.3	3.9	3.0
	Mean intra-scanner	7.5	0.1	1.3		7.6	0.1	1.3	
Cingulum	Inter-scanner	7.6	0.4	5.3	4.1	7.5	0.4	5.3	4.1
	Mean intra-scanner	7.6	0.1	1.3		7.5	0.1	1.3	
Posterior limb internal capsule	Inter-scanner	7.2	0.5	6.9	4.9	7.0	0.4	5.7	4.1
	Mean intra-scanner	7.2	0.1	1.4		7.0	0.1	1.4	
Sagittal stratum	Inter-scanner	8.3	0.5	6.0	2.5	8.1	0.4	4.9	4.1
	Mean intra-scanner	8.3	0.2	2.4		8.1	0.1	1.2	
Uncinate fasciculus	Inter-scanner	8.0	0.4	5.0	2.0	8.1	0.3	3.7	1.5
	Mean intra-scanner	8.1	0.2	2.5		8.1	0.2	2.5	

**BILATERAL**									
Corpus callosum	Inter-scanner	9.0	0.6	6.7	6.1				
	Mean intra-scanner	9.0	0.1	1.1					

*All scans were included (*n* = 24). DTI data were not available Scanner B. MD is scaled × 10,000 mm^2^/s.*

**Table:** **Members of the Tuberous Sclerosis Autism Center of Excellence Research Network (TACERN)**.

**Member name**	**Affiliation**	**COIs/Disclosures**
Simon K. Warfield, Ph.D.	Department of Radiology, Boston Children’s Hospital, Boston, MA	None
Jurriaan M. Peters, MD, Ph.D.	Division of Epilepsy and Clinical Neurophysiology, Department of Neurology, Boston Children’s Hospital, Boston, MA	None
Monisha Goyal, MD	Department of Neurology, University of Alabama at Birmingham, Birmingham, AL	
Deborah A. Pearson, Ph.D.	Department of Psychiatry and Behavioral Sciences, McGovern Medical School, University of Texas Health Science Center at Houston, Houston, TX	Curemark LLC–Consulting fees, Research grants and Travel Reimbursement — — — — — — — — — — — — — — — — — — — - *In the last year, my team has also done psych consults for Dr. Northrup’s Biomarin clinical trial and Dr. Koenig’s Novartis clinical trial. Thus, although I am listing myself as having received research grant funds from these projects, the funds are actually awarded to Hope and Mary Kay*. Biomarin: Research grant funds (Northrup, PI) Novartis: Research grant funds (Koenig, PI)
Marian E. Williams, Ph.D.	Keck School of Medicine of USC, University of Southern California, Los Angeles, California	None
Ellen Hanson, Ph.D.	Department of Developmental Medicine, Boston Children’s Hospital, Boston, MA	None
Nicole Bing, Psy.D.	Department of Developmental and Behavioral Pediatrics, Cincinnati Children’s Hospital Medical Center, Cincinnati, Ohio	
Bridget Kent, MA, CCC-SLP	Department of Developmental and Behavioral Pediatrics, Cincinnati Children’s Hospital Medical Center, Cincinnati, Ohio	
Sarah O’Kelley, Ph.D.	University of Alabama at Birmingham, Birmingham, AL	
Rajna Filip-Dhima, MS	Department of Neurology, Boston Children’s Hospital, Boston, MA	None
Kira Dies, ScM, CGC	Department of Neurology, Boston Children’s Hospital, Boston, MA	None
Stephanie Bruns	Cincinnati Children’s Hospital Medical Center, Cincinnati, OH	
Benoit Scherrer, Ph.D.	Department of Radiology, Boston Children’s Hospital, Boston, MA	
Gary Cutter, Ph.D.	University of Alabama at Birmingham, Data Coordinating Center, Birmingham, AL	
Donna S. Murray, Ph.D.	Autism Speaks	None
Steven L. Roberds, Ph.D.	Tuberous Sclerosis Alliance	Research funding from Novartis

Inter-scanner FA CVs were less than 5% in 12 of 17 labels evaluated and between 5 and 8% for 5 of 17 labels, including bilateral arcuate fasciculus region 3, left sagittal stratum, and right posterior limb internal capsule and uncinate fasciculus. Inter-scanner MD CVs were less than 5% in 7 of 17 labels evaluated. MD inter-scanner CV was maximal in left and right anterior limb of the internal capsule, at 8.2 and 8.1%, respectively. The least variable FA across scanners was the right arcuate fasciculus region 1 at 2.4%, while the least variable MD CV across scanners was the right arcuate fasciculus region 2.0 at 2.7%.

The FA of the corpus callosum and left and right posterior limbs of the internal capsules had the lowest average intra-scanner CV, at 1.7%, whereas the right uncinate fasciculus had the highest average intra-scanner FA CV, at 5.3%, driven by an intra-scanner CV of 10.3% on scanner E. The MD of corpus callosum had the lowest average intra-scanner CV at 1.1 %, and MD of the left and right uncinate fasciculus had the highest intra-scanner MD CV on average, at 2.5%.

The inter-scanner FA CV exceeded the mean intra-scanner FA CV by a factor of 1.9 on average and ranged from a factor of 1.0–3.0. The inter-scanner MD CV exceeded the mean intra-scanner MD CV by a factor of 3.8 on average, and ranged from a factor of 1.5–6.1.

## Discussion

We evaluated the reproducibility of MRI data of the ACR phantom and a traveling human phantom from seven scanners across 5 sites in a multi-site imaging study over a period of 5 years. Scanners are often subjected to system maintenance upgrades over time, and the hardware for imaging can be heterogeneous across centers. Analyzing the reproducibility of imaging measures across scanners is therefore important when combining measures from different scanners into a single dataset.

Our methods include reproducibility analyses of (1) signal intensity and uniformity using T1w images of the ACR phantom, (2) brain segmentation label volumes in a human volunteer, and (3) DTI metrics of white matter labels in a human volunteer within and across scanners used in the TACERN study. Analysis of signal intensity and uniformity demonstrate that SNR was consistent over time, with a CV of less than 2.1% in 5 of 7 scanners over time. Two scanners that underwent software upgrades demonstrated the highest SNR CV of 9.9 and 5.8%. SNR is influenced by a number of scanner-related factors, including resonance frequency, transmitter gain, scan acceleration, and coil loading (Keenan et al., 2018), any of which could vary with a software upgrade. Image uniformity on all scanners exceeded the ACR recommended IU of 82% or higher on 3T systems (American College of Radiology, 2018). IU was 92% on average across scanners, in line with reports of ACR IU in previous quality assurance studies (Chen et al., 2004; Davids et al., 2014). Variation in IU can be due to many factors, including but not limited to B_0_ and B_1_ non-uniformities, gradient linearity, and eddy currents (Keenan et al., 2018). Scanner C exhibited two temporally segregated clusters of IU, indicating an initial non-uniformity that was later corrected.

We found the inter-scanner variability of brain volume measurements overall was low and in line with other multisite studies of brain volume measurements. We found inter-scanner volume CV was on average 3.3%, ranged from 1.4 to 9.9%, and was less than 5% in 35 of 38 labels. Previous studies generally report average inter-scanner CV of less than 5%, depending on the brain structure analyzed (Huppertz et al., 2010; De Guio et al., 2016), and also have found a similarly high CSF inter-scanner CV of 9% (Huppertz et al., 2010). We found mean intra-scanner volume CV was on average 1.1% and ranged from 0.5 to 3.0%, similar to previous studies that report 0–3% intra-scanner CV of tissue volumes (de Boer et al., 2010; Huppertz et al., 2010; Landman et al., 2011; Maclaren et al., 2014; De Guio et al., 2016). Despite variable SNR on scanner E over the study period, scanner E volume measurements were not outlying from the rest of the data set, likely due to the robustness of the automated brain segmentation methodology.

Inter-scanner label volume CV was on average 2.5 times more variable than intra-scanner label volume CV. Higher inter-scanner compared to intra-scanner CV is expected given variation in hardware and software across scanners, in addition to intra-scanner sources of variance including noise and subject positioning within the scanner. Within-subject biological sources of variation also contribute to inter-scanner measurement variation. Previous work has shown that time of day and level of hydration affects brain and cerebrospinal fluid volume measurements (Dieleman et al., 2017).

We found the reproducibility of DTI measurements within and across TACERN scanners is in accordance with previous studies of multisite DTI studies. Over all white matter labels, we found intra-scanner FA (2.5%) was greater than the intra-scanner MD (1.5%). Our findings are in line with past studies that generally report <3% CV FA (Heiervang et al., 2006; Zhu et al., 2012; Grech-Sollars et al., 2015; Acheson et al., 2017; Palacios et al., 2017) . Reports of MD are more variable, ranging from 0 to 7 % with most studies clustering around 2% intra-scanner CV MD (Heiervang et al., 2006; Magnotta et al., 2012; Grech-Sollars et al., 2015; Shahim et al., 2017; Nencka et al., 2018; Zhou et al., 2018).

We found an inter-scanner FA CV of 4.5%, in line with past studies of inter-scanner variability in white matter ROIs that report <5% CV for FA (Pagani et al., 2010; Vollmar et al., 2010; Grech-Sollars et al., 2015; Nencka et al., 2018). Studies of inter-scanner variability of FA within larger ROIs, such as whole brain white matter, lobar white matter, or white matter tracts generally report a CV of less than 4% (Magnotta et al., 2012; Grech-Sollars et al., 2015). For MD, we found an inter-scanner CV of 5.4%, greater than the inter-scanner FA CV. In contrast, past studies typically report an inter-scanner MD CV of <3%, lower than inter-scanner FA CV (Pagani et al., 2010; Magnotta et al., 2012; Grech-Sollars et al., 2015; Palacios et al., 2017; Nencka et al., 2018; Zhou et al., 2018). We found the average ratio of inter- to intra-scanner CV FA was approximately 2 to 1; whereas the average inter- to intra-scanner CV MD ratio was approximately 4 to 1. Thus, our data suggest that the FA is more robust to inter-scanner variations than MD.

This study is limited because scan-rescan was not possible on all study scanners due to scheduling demands of the clinical scanners utilized in the TACERN study. Thus change in subject anatomy over time is an additional source of measurement error that cannot be excluded from the intra-scanner CV metric.

## Conclusion

Volumetric and DTI measurements acquired on TACERN study scanners are highly reproducible between and within scanners. Our findings will be useful for calculating sample sizes needed to identify group differences corresponding to pre-specified effect sizes, and for interpreting future MRI findings in the TACERN study.

## Ethics Statement

All study procedures were approved by the Institutional Review Board at BCH, CCHMC, UAB, UCLA, and UTH, and the human phantom provided written informed consent.

## Author Contributions

AP, BS, CV-A, JP, EB, DK, HN, JW, MS, SP, and SW conceived and designed the study. All authors collected and analyzed the data. KK, AP, BS, RF-D, XT-F, JP, MS, and SW drafted a significant portion of the manuscript.

## Conflict of Interest Statement

JW has received research funding from the Novartis and GW Pharmaceutical, and is an editorial board member of the journal Pediatric Investigation. DK has received research funding and consulting fees from the Novartis Pharmaceuticals, and additional consulting fees from the Mallinckrodt Pharmaceuticals, AXIS Media, and Advance Medical. MS has received research funding from the Roche, Novartis, Pfizer, LAM Therapeutics, Rugen, Ibsen, and Neuren and has served on the Scientific Advisory Board of Sage Therapeutics, Roche, and Takeda. The reviewer KH declared a shared affiliation, with no collaboration, with several of the authors [AP, BS, XT-F, RF-D, KK, CV-A, SC, EC, MD, MV, SP, JP, MS, SW], to the handling Editor at the time of review. The remaining authors declare that the research was conducted in the absence of any commercial or financial relationships that could be construed as a potential conflict of interest.

## Footnotes

1 http://crl.med.harvard.edu

2 http://www.neuromorphometrics.org

## Abbreviations

ACR: American College of Radiology
ASD: autism spectrum disorders
BCH: Boston Children’s Hospital
CCHMC: Cincinnati Children’s Hospital Medical Center
CUSP: cube and sphere
CV: coefficient of variation
DTI: diffusion tensor imaging
DWI: diffusion weighted imaging
FA: fractional anisotropy
FOV: field of view
GE: General Electric
ICC: intracranial cavity
IU: integral uniformity
MD: mean diffusivity
MRI: magnetic resonance imaging
PSTAPLE: probabilistic simultaneous truth and performance level estimation
ROI: region of interest
SD: standard deviation
SNR: signal to noise ratio
T1w: T1-weighted
T2w: T2-weighted
TACERN: Tuberous Sclerosis Complex Autism Center of Excellence Research Network
TE: echo time
TR: repetition time
TSC: tuberous sclerosis complex
UAB: University of Alabama
UCLA: University of California Los Angeles
UTH: University of Texas Houston.
