# Supplementary material for: Reproducibility of Structural and Diffusion Tensor Imaging in the TACERN Multi-Center Study
Source: Front Integr Neurosci. 2019 Jul 17;13:24. doi: 10.3389/fnint.2019.00024 (PMC6650594; doi:10.3389/fnint.2019.00024)
Supplement: Supplementary file 1 [file Image_1.pdf]

Supplemental Figure1.

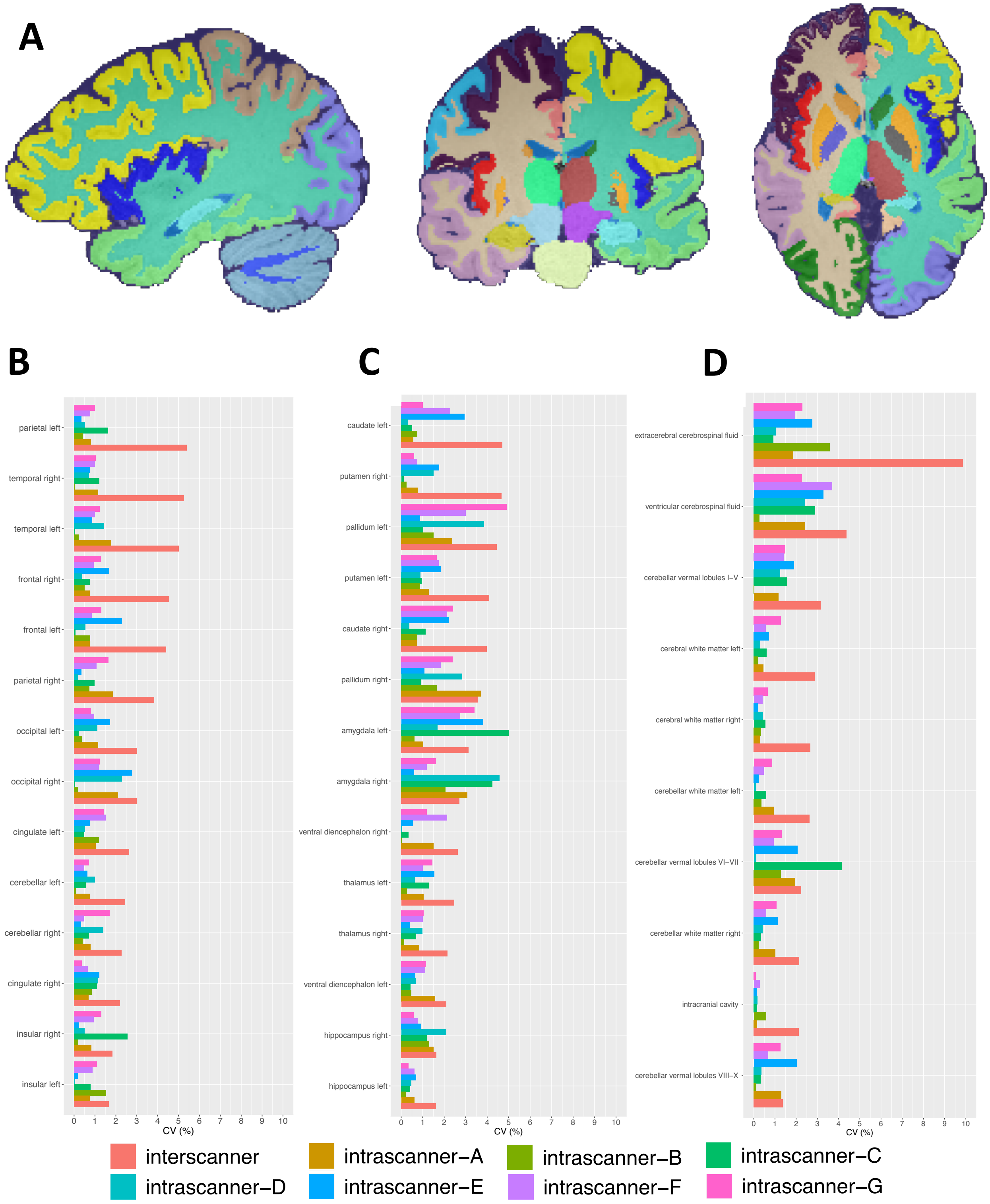

Supplemental Figure 1.
Sagittal, coronal, and axial views of a fully automatic brain parcellation result. Each color label identifies a brain structure of interest. B. Intra and Interscanner coefficient of variation of brain parcellation cortical label volume. Labels are ordered from bottom to top by increasing interscanner coefficient of variation. C. Intra and Interscanner coefficient of variation of brain parcellation subcortical label volumes. Labels are ordered from bottom to top by increasing interscanner coefficient of variation. D. Intra and Interscanner coefficient of variation of brain parcellation non-cortical and non-subcortical label volumes. Labels are ordered from bottom to top by increasing interscanner coefficient of variation.
