# Supplementary material for: Reproducibility of Structural and Diffusion Tensor Imaging in the TACERN Multi-Center Study
Source: Front Integr Neurosci. 2019 Jul 17;13:24. doi: 10.3389/fnint.2019.00024 (PMC6650594; doi:10.3389/fnint.2019.00024)
Supplement: Supplementary file 2 [file Image_2.pdf]

Supplemental Figure 2.

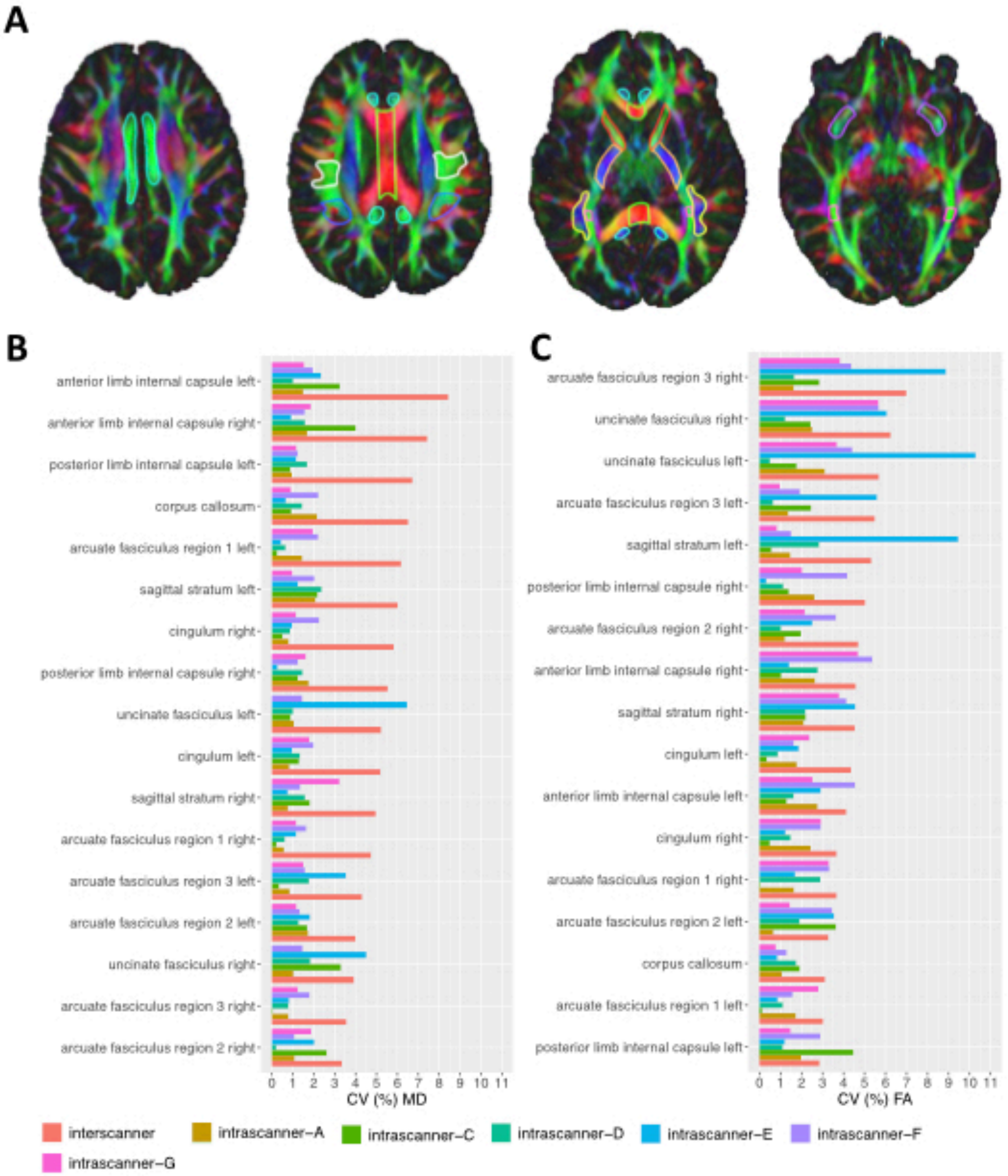

Supplemental Figure 2.
A. White matter regions of interest (ROI) superimposed on a color map of the principal diffusion directions. Red color map voxels indicate left-right diffusion, green color map voxels indicate anterior-posterior diffusion, blue color map voxels indicate inferior-superior diffusion, and other colors indicate intermediate diffusion directions. Four axial slices from a single scan depict 2D slices of 3D white matter ROI, outlined in unique colors: light blue=cingulum, green=corpus callosum, white=arcuate fasciculus region 1; royal blue = arcuate fasciculus region 2, red=anterior limb of the internal capsule, orange = posterior limb of the internal capsule, yellow = arcuate fasciculus region 3, pink = sagittal stratum, and purple=uncinate fasciculus. B. Intra and Interscanner coefficient of variation of white matter ROI mean diffusivity (MD). Labels are ordered from bottom to top by increasing interscanner coefficient of variation. C. Intra and Interscanner coefficient of variation of white matter ROI fractional anisotropy (FA). Labels are ordered from bottom to top by increasing interscanner coefficient of variation.
