# Supplementary material for: Reproducibility of Structural and Diffusion Tensor Imaging in the TACERN Multi-Center Study
Source: Front Integr Neurosci. 2019 Jul 17;13:24. doi: 10.3389/fnint.2019.00024 (PMC6650594; doi:10.3389/fnint.2019.00024)
Supplement: Supplementary file 3 [file Table_1.DOCX]

Supplemental Table 1. Inter and intra-scanner variability of brain parcellation label volumes. All scans were included (n=26).

| Bilateral Labels |  |  | LEFT |  |  |  | RIGHT |  |
| --- | --- | --- | --- | --- | --- | --- | --- | --- |
|  |  | **Mean (mm^3^)** | **SD (mm^3^)** | **CV (%)** |  | **Mean (mm^3^)** | **SD (mm^3^)** | **CV (%)** |
| cerebellar cortex | inter-scanner | 51274 | 1258 | 2.45 |  | 51490 | 1171 | 2.27 |
|  | intra-scanner-A | 50233 | 377 | 0.75 |  | 51031 | 401 | 0.79 |
|  | intra-scanner-B | 52536 | 49 | 0.09 |  | 52808 | 218 | 0.41 |
|  | intra-scanner-C | 50091 | 281 | 0.56 |  | 50731 | 361 | 0.71 |
|  | intra-scanner-D | 50199 | 501 | 1.00 |  | 50038 | 698 | 1.39 |
|  | intra-scanner-E | 51147 | 326 | 0.64 |  | 51090 | 175 | 0.34 |
|  | intra-scanner-F | 51006 | 252 | 0.49 |  | 51268 | 234 | 0.46 |
|  | intra-scanner-G | 53675 | 380 | 0.71 |  | 53548 | 912 | 1.70 |
| cingulate cortex | inter-scanner | 12266 | 324 | 2.64 |  | 10869 | 238 | 2.19 |
|  | intra-scanner-A | 12439 | 128 | 1.03 |  | 10903 | 76 | 0.70 |
|  | intra-scanner-B | 13135 | 156 | 1.19 |  | 11532 | 98 | 0.85 |
|  | intra-scanner-C | 12447 | 58 | 0.46 |  | 10892 | 119 | 1.10 |
|  | intra-scanner-D | 12121 | 65 | 0.53 |  | 10963 | 127 | 1.16 |
|  | intra-scanner-E | 11982 | 90 | 0.75 |  | 10595 | 128 | 1.20 |
|  | intra-scanner-F | 12154 | 185 | 1.52 |  | 10825 | 71 | 0.66 |
|  | intra-scanner-G | 12156 | 172 | 1.41 |  | 10772 | 40 | 0.37 |
| frontal cortex | inter-scanner | 94435 | 4163 | 4.41 |  | 96357 | 4384 | 4.55 |
|  | intra-scanner-A | 92304 | 693 | 0.75 |  | 93046 | 695 | 0.75 |
|  | intra-scanner-B | 91712 | 710 | 0.77 |  | 93931 | 469 | 0.50 |
|  | intra-scanner-C | 87712 | 58 | 0.07 |  | 89420 | 670 | 0.75 |
|  | intra-scanner-D | 95965 | 521 | 0.54 |  | 98434 | 384 | 0.39 |
|  | intra-scanner-E | 94833 | 2174 | 2.29 |  | 96164 | 1622 | 1.69 |
|  | intra-scanner-F | 92870 | 787 | 0.85 |  | 95301 | 895 | 0.94 |
|  | intra-scanner-G | 102485 | 1342 | 1.31 |  | 104832 | 1337 | 1.28 |
| insular cortex | inter-scanner | 6356 | 106 | 1.67 |  | 6773 | 124 | 1.83 |
|  | intra-scanner-A | 6334 | 48 | 0.75 |  | 6782 | 57 | 0.83 |
|  | intra-scanner-B | 6574 | 101 | 1.54 |  | 6993 | 14 | 0.20 |
|  | intra-scanner-C | 6396 | 51 | 0.79 |  | 6931 | 177 | 2.55 |
|  | intra-scanner-D | 6462 | 3 | 0.05 |  | 6887 | 34 | 0.50 |
|  | intra-scanner-E | 6331 | 12 | 0.18 |  | 6672 | 16 | 0.24 |
|  | intra-scanner-F | 6350 | 56 | 0.88 |  | 6741 | 63 | 0.94 |
|  | intra-scanner-G | 6203 | 68 | 1.10 |  | 6650 | 87 | 1.31 |
| occipital cortex | inter-scanner | 33598 | 1015 | 3.02 |  | 36392 | 1090 | 3.00 |
|  | intra-scanner-A | 32814 | 377 | 1.15 |  | 35851 | 757 | 2.11 |
|  | intra-scanner-B | 33268 | 122 | 0.37 |  | 36065 | 70 | 0.19 |
|  | intra-scanner-C | 32601 | 73 | 0.22 |  | 34889 | 18 | 0.05 |
|  | intra-scanner-D | 34512 | 384 | 1.11 |  | 36439 | 837 | 2.30 |
|  | intra-scanner-E | 33450 | 577 | 1.72 |  | 36150 | 1003 | 2.77 |
|  | intra-scanner-F | 33056 | 322 | 0.97 |  | 36217 | 426 | 1.18 |
|  | intra-scanner-G | 35456 | 283 | 0.80 |  | 38358 | 473 | 1.23 |
| parietal cortex | inter-scanner | 48671 | 2627 | 5.40 |  | 47279 | 1812 | 3.83 |
|  | intra-scanner-A | 46321 | 375 | 0.81 |  | 45485 | 842 | 1.85 |
|  | intra-scanner-B | 48938 | 204 | 0.42 |  | 48292 | 357 | 0.74 |
|  | intra-scanner-C | 43953 | 712 | 1.62 |  | 44425 | 433 | 0.98 |
|  | intra-scanner-D | 48539 | 255 | 0.53 |  | 47245 | 91 | 0.19 |
|  | intra-scanner-E | 49544 | 177 | 0.36 |  | 47502 | 171 | 0.36 |
|  | intra-scanner-F | 48029 | 369 | 0.77 |  | 46840 | 507 | 1.08 |
|  | intra-scanner-G | 53594 | 536 | 1.00 |  | 50563 | 833 | 1.65 |
| temporal cortex | inter-scanner | 61785 | 3102 | 5.02 |  | 61533 | 3238 | 5.26 |
|  | intra-scanner-A | 57994 | 1027 | 1.77 |  | 57404 | 663 | 1.16 |
|  | intra-scanner-B | 62794 | 141 | 0.22 |  | 62466 | 18 | 0.03 |
|  | intra-scanner-C | 56881 | 22 | 0.04 |  | 56222 | 682 | 1.21 |
|  | intra-scanner-D | 60879 | 878 | 1.44 |  | 61326 | 434 | 0.71 |
|  | intra-scanner-E | 63063 | 548 | 0.87 |  | 61499 | 467 | 0.76 |
|  | intra-scanner-F | 61669 | 612 | 0.99 |  | 62028 | 622 | 1.00 |
|  | intra-scanner-G | 67125 | 816 | 1.22 |  | 67175 | 701 | 1.04 |
| amygdala | inter-scanner | 1307 | 41 | 3.14 |  | 1289 | 35 | 2.71 |
|  | intra-scanner-A | 1320 | 14 | 1.03 |  | 1270 | 39 | 3.08 |
|  | intra-scanner-B | 1352 | 8 | 0.61 |  | 1317 | 27 | 2.07 |
|  | intra-scanner-C | 1288 | 64 | 5.00 |  | 1243 | 53 | 4.25 |
|  | intra-scanner-D | 1340 | 23 | 1.70 |  | 1266 | 58 | 4.58 |
|  | intra-scanner-E | 1266 | 48 | 3.81 |  | 1298 | 8 | 0.60 |
|  | intra-scanner-F | 1305 | 36 | 2.75 |  | 1295 | 15 | 1.19 |
|  | intra-scanner-G | 1297 | 44 | 3.41 |  | 1317 | 21 | 1.62 |
| caudate | inter-scanner | 4130 | 195 | 4.71 |  | 4270 | 170 | 3.99 |
|  | intra-scanner-A | 4330 | 25 | 0.57 |  | 4454 | 33 | 0.73 |
|  | intra-scanner-B | 4223 | 31 | 0.74 |  | 4431 | 33 | 0.74 |
|  | intra-scanner-C | 4328 | 22 | 0.50 |  | 4441 | 50 | 1.13 |
|  | intra-scanner-D | 4140 | 13 | 0.30 |  | 4270 | 16 | 0.38 |
|  | intra-scanner-E | 3874 | 114 | 2.95 |  | 4066 | 89 | 2.20 |
|  | intra-scanner-F | 3966 | 91 | 2.29 |  | 4123 | 88 | 2.14 |
|  | intra-scanner-G | 4320 | 43 | 1.00 |  | 4383 | 106 | 2.41 |
| hippocampus | inter-scanner | 4038 | 65 | 1.62 |  | 3984 | 65 | 1.63 |
|  | intra-scanner-A | 4034 | 25 | 0.61 |  | 3975 | 60 | 1.51 |
|  | intra-scanner-B | 4222 | 9 | 0.21 |  | 4122 | 54 | 1.30 |
|  | intra-scanner-C | 4037 | 17 | 0.41 |  | 3986 | 47 | 1.19 |
|  | intra-scanner-D | 4064 | 19 | 0.47 |  | 4038 | 84 | 2.09 |
|  | intra-scanner-E | 4054 | 28 | 0.69 |  | 3987 | 38 | 0.94 |
|  | intra-scanner-F | 4012 | 25 | 0.62 |  | 3943 | 30 | 0.76 |
|  | intra-scanner-G | 3963 | 14 | 0.35 |  | 3947 | 23 | 0.58 |
| pallidum | inter-scanner | 1610 | 72 | 4.45 |  | 1666 | 59 | 3.56 |
|  | intra-scanner-A | 1638 | 39 | 2.37 |  | 1591 | 59 | 3.71 |
|  | intra-scanner-B | 1760 | 26 | 1.50 |  | 1789 | 30 | 1.66 |
|  | intra-scanner-C | 1605 | 16 | 1.02 |  | 1636 | 15 | 0.92 |
|  | intra-scanner-D | 1553 | 60 | 3.86 |  | 1681 | 48 | 2.83 |
|  | intra-scanner-E | 1585 | 14 | 0.87 |  | 1698 | 18 | 1.08 |
|  | intra-scanner-F | 1566 | 47 | 3.00 |  | 1668 | 31 | 1.84 |
|  | intra-scanner-G | 1655 | 81 | 4.91 |  | 1650 | 39 | 2.39 |
| putamen | inter-scanner | 5432 | 222 | 4.09 |  | 5274 | 246 | 4.66 |
|  | intra-scanner-A | 5323 | 69 | 1.29 |  | 5219 | 40 | 0.77 |
|  | intra-scanner-B | 5484 | 48 | 0.87 |  | 5395 | 14 | 0.25 |
|  | intra-scanner-C | 5390 | 51 | 0.95 |  | 5315 | 6 | 0.11 |
|  | intra-scanner-D | 5437 | 48 | 0.89 |  | 5260 | 79 | 1.51 |
|  | intra-scanner-E | 5244 | 96 | 1.83 |  | 5040 | 89 | 1.76 |
|  | intra-scanner-F | 5335 | 93 | 1.74 |  | 5111 | 38 | 0.74 |
|  | intra-scanner-G | 5891 | 98 | 1.66 |  | 5776 | 34 | 0.60 |
| thalamus | inter-scanner | 7903 | 194 | 2.46 |  | 7479 | 161 | 2.16 |
|  | intra-scanner-A | 8013 | 83 | 1.04 |  | 7580 | 64 | 0.84 |
|  | intra-scanner-B | 8310 | 22 | 0.26 |  | 7818 | 11 | 0.14 |
|  | intra-scanner-C | 7949 | 102 | 1.29 |  | 7614 | 53 | 0.70 |
|  | intra-scanner-D | 7869 | 49 | 0.63 |  | 7385 | 73 | 0.99 |
|  | intra-scanner-E | 7664 | 118 | 1.54 |  | 7326 | 29 | 0.40 |
|  | intra-scanner-F | 7785 | 78 | 1.00 |  | 7358 | 74 | 1.01 |
|  | intra-scanner-G | 8040 | 116 | 1.45 |  | 7578 | 79 | 1.04 |
| ventral diencephalon | inter-scanner | 5592 | 117 | 2.09 |  | 5538 | 146 | 2.64 |
|  | intra-scanner-A | 5623 | 89 | 1.58 |  | 5616 | 85 | 1.51 |
|  | intra-scanner-B | 5807 | 27 | 0.47 |  | 5763 | 1 | 0.02 |
|  | intra-scanner-C | 5567 | 25 | 0.44 |  | 5557 | 19 | 0.34 |
|  | intra-scanner-D | 5695 | 38 | 0.67 |  | 5688 | 3 | 0.05 |
|  | intra-scanner-E | 5504 | 36 | 0.66 |  | 5561 | 30 | 0.54 |
|  | intra-scanner-F | 5480 | 61 | 1.11 |  | 5472 | 117 | 2.14 |
|  | intra-scanner-G | 5672 | 65 | 1.15 |  | 5320 | 64 | 1.20 |
| cerebellar white matter | inter-scanner | 18600 | 487 | 2.62 |  | 18438 | 394 | 2.14 |
|  | intra-scanner-A | 18888 | 177 | 0.94 |  | 18438 | 188 | 1.02 |
|  | intra-scanner-B | 19956 | 71 | 0.36 |  | 19603 | 46 | 0.23 |
|  | intra-scanner-C | 18325 | 106 | 0.58 |  | 18296 | 62 | 0.34 |
|  | intra-scanner-D | 17963 | 20 | 0.11 |  | 17993 | 76 | 0.42 |
|  | intra-scanner-E | 18326 | 43 | 0.23 |  | 18459 | 208 | 1.13 |
|  | intra-scanner-F | 18552 | 89 | 0.48 |  | 18409 | 109 | 0.59 |
|  | intra-scanner-G | 18610 | 160 | 0.86 |  | 18289 | 197 | 1.08 |
| cerebral white matter | inter-scanner | 245773 | 7068 | 2.88 |  | 249787 | 6668 | 2.67 |
|  | intra-scanner-A | 244980 | 1124 | 0.46 |  | 248109 | 774 | 0.31 |
|  | intra-scanner-B | 261363 | 515 | 0.20 |  | 264806 | 921 | 0.35 |
|  | intra-scanner-C | 239321 | 1451 | 0.61 |  | 243084 | 1313 | 0.54 |
|  | intra-scanner-D | 241280 | 737 | 0.31 |  | 245800 | 1086 | 0.44 |
|  | intra-scanner-E | 244332 | 1768 | 0.72 |  | 249479 | 475 | 0.19 |
|  | intra-scanner-F | 240820 | 1341 | 0.56 |  | 245049 | 998 | 0.41 |
|  | intra-scanner-G | 255477 | 3281 | 1.28 |  | 258894 | 1727 | 0.67 |

| Whole brain Labels |  | Mean (mm^3^) | SD (mm^3^) | CV (%) |
| --- | --- | --- | --- | --- |
| Intracranial cavity | inter-scanner | 1553414 | 33165 | 2.13 |
|  | intra-scanner-A | 1541517 | 2530 | 0.16 |
|  | intra-scanner-B | 1661311 | 9587 | 0.58 |
|  | intra-scanner-C | 1540745 | 2478 | 0.16 |
|  | intra-scanner-D | 1541193 | 2806 | 0.18 |
|  | intra-scanner-E | 1559737 | 2229 | 0.14 |
|  | intra-scanner-F | 1534694 | 4300 | 0.28 |
|  | intra-scanner-G | 1553301 | 1512 | 0.10 |
| extracerebral cerebrospinal fluid | inter-scanner | 262844 | 25930 | 9.87 |
|  | intra-scanner-A | 271521 | 5057 | 1.86 |
|  | intra-scanner-B | 302703 | 10878 | 3.59 |
|  | intra-scanner-C | 292538 | 2720 | 0.93 |
|  | intra-scanner-D | 262600 | 2697 | 1.03 |
|  | intra-scanner-E | 269936 | 7454 | 2.76 |
|  | intra-scanner-F | 263686 | 5141 | 1.95 |
|  | intra-scanner-G | 211007 | 4848 | 2.30 |
| ventricular cerebrospinal fluid | inter-scanner | 19614 | 858 | 4.37 |
|  | intra-scanner-A | 19732 | 479 | 2.43 |
|  | intra-scanner-B | 21919 | 58 | 0.27 |
|  | intra-scanner-C | 19406 | 561 | 2.89 |
|  | intra-scanner-D | 19222 | 466 | 2.43 |
|  | intra-scanner-E | 19480 | 639 | 3.28 |
|  | intra-scanner-F | 19347 | 713 | 3.69 |
|  | intra-scanner-G | 19340 | 438 | 2.27 |
| cerebellar vermal lobules I-V | inter-scanner | 4699 | 148 | 3.16 |
|  | intra-scanner-A | 4725 | 55 | 1.16 |
|  | intra-scanner-B | 4775 | 1 | 0.02 |
|  | intra-scanner-C | 4737 | 74 | 1.56 |
|  | intra-scanner-D | 4632 | 58 | 1.25 |
|  | intra-scanner-E | 4591 | 87 | 1.89 |
|  | intra-scanner-F | 4585 | 65 | 1.41 |
|  | intra-scanner-G | 4972 | 74 | 1.48 |
| cerebellar vermal lobules VI-VII | inter-scanner | 1530 | 34 | 2.23 |
|  | intra-scanner-A | 1544 | 30 | 1.96 |
|  | intra-scanner-B | 1565 | 20 | 1.28 |
|  | intra-scanner-C | 1523 | 63 | 4.14 |
|  | intra-scanner-D | 1491 | 2 | 0.11 |
|  | intra-scanner-E | 1543 | 32 | 2.06 |
|  | intra-scanner-F | 1547 | 15 | 0.94 |
|  | intra-scanner-G | 1492 | 20 | 1.32 |
| cerebellar vermal lobules VIII-X | inter-scanner | 2938 | 40 | 1.38 |
|  | intra-scanner-A | 2923 | 38 | 1.30 |
|  | intra-scanner-B | 2957 | 3 | 0.10 |
|  | intra-scanner-C | 2931 | 10 | 0.33 |
|  | intra-scanner-D | 2924 | 11 | 0.36 |
|  | intra-scanner-E | 2957 | 60 | 2.02 |
|  | intra-scanner-F | 2965 | 20 | 0.68 |
|  | intra-scanner-G | 2888 | 36 | 1.26 |

CV=coefficient of variation.

Supplemental Table 2. Inter and intra-scanner variability of FA in white matter ROIs. All scans were included (n=24). DTI data were not available for Scanner B.

|  |  | LEFT | | |  | | RIGHT | | | |
| --- | --- | --- | --- | --- | --- | --- | --- | --- | --- | --- |
|  |  | Mean FA | SD FA | CV(%) | |  | | Mean FA | SD FA | CV(%) |
| anterior limb internal capsule | inter-scanner | 0.509 | 0.021 | 4.12 | |  | | 0.521 | 0.024 | 4.57 |
|  | intra-scanner-A | 0.493 | 0.014 | 2.75 | |  | | 0.496 | 0.013 | 2.63 |
|  | intra-scanner-C | 0.524 | 0.007 | 1.28 | |  | | 0.527 | 0.005 | 1.03 |
|  | intra-scanner-D | 0.513 | 0.008 | 1.62 | |  | | 0.512 | 0.014 | 2.77 |
|  | intra-scanner-E | 0.505 | 0.015 | 2.91 | |  | | 0.533 | 0.008 | 1.41 |
|  | intra-scanner-F | 0.500 | 0.023 | 4.54 | |  | | 0.518 | 0.028 | 5.36 |
|  | intra-scanner-G | 0.536 | 0.013 | 2.52 | |  | | 0.542 | 0.025 | 4.68 |
| arcuate fasciculus region 1 | inter-scanner | 0.412 | 0.012 | 3.02 | |  | | 0.434 | 0.016 | 3.66 |
|  | intra-scanner-A | 0.401 | 0.007 | 1.72 | |  | | 0.419 | 0.007 | 1.63 |
|  | intra-scanner-C | 0.433 | 0.001 | 0.12 | |  | | 0.437 | 0.000 | 0.04 |
|  | intra-scanner-D | 0.408 | 0.005 | 1.11 | |  | | 0.422 | 0.012 | 2.89 |
|  | intra-scanner-E | 0.409 | 0.004 | 0.86 | |  | | 0.444 | 0.008 | 1.69 |
|  | intra-scanner-F | 0.407 | 0.006 | 1.57 | |  | | 0.429 | 0.014 | 3.31 |
|  | intra-scanner-G | 0.428 | 0.012 | 2.80 | |  | | 0.453 | 0.015 | 3.29 |
| arcuate fasciculus region 2 | inter-scanner | 0.380 | 0.012 | 3.27 | |  | | 0.415 | 0.019 | 4.69 |
|  | intra-scanner-A | 0.372 | 0.002 | 0.65 | |  | | 0.395 | 0.005 | 1.20 |
|  | intra-scanner-C | 0.388 | 0.014 | 3.63 | |  | | 0.418 | 0.008 | 1.97 |
|  | intra-scanner-D | 0.368 | 0.007 | 1.90 | |  | | 0.400 | 0.004 | 1.02 |
|  | intra-scanner-E | 0.379 | 0.013 | 3.54 | |  | | 0.428 | 0.011 | 2.51 |
|  | intra-scanner-F | 0.379 | 0.013 | 3.45 | |  | | 0.409 | 0.015 | 3.63 |
|  | intra-scanner-G | 0.393 | 0.006 | 1.44 | |  | | 0.444 | 0.010 | 2.15 |
| arcuate fasciculus region 3 | inter-scanner | 0.456 | 0.025 | 5.48 | |  | | 0.396 | 0.028 | 6.98 |
|  | intra-scanner-A | 0.438 | 0.006 | 1.36 | |  | | 0.378 | 0.006 | 1.62 |
|  | intra-scanner-C | 0.461 | 0.011 | 2.45 | |  | | 0.398 | 0.011 | 2.84 |
|  | intra-scanner-D | 0.429 | 0.003 | 0.64 | |  | | 0.390 | 0.006 | 1.65 |
|  | intra-scanner-E | 0.499 | 0.028 | 5.58 | |  | | 0.439 | 0.039 | 8.86 |
|  | intra-scanner-F | 0.451 | 0.009 | 1.92 | |  | | 0.382 | 0.017 | 4.36 |
|  | intra-scanner-G | 0.458 | 0.004 | 0.97 | |  | | 0.400 | 0.015 | 3.82 |
| cingulum | inter-scanner | 0.455 | 0.020 | 4.35 | |  | | 0.452 | 0.017 | 3.66 |
|  | intra-scanner-A | 0.421 | 0.007 | 1.77 | |  | | 0.430 | 0.010 | 2.43 |
|  | intra-scanner-C | 0.448 | 0.002 | 0.34 | |  | | 0.449 | 0.002 | 0.49 |
|  | intra-scanner-D | 0.469 | 0.004 | 0.87 | |  | | 0.450 | 0.007 | 1.48 |
|  | intra-scanner-E | 0.450 | 0.008 | 1.86 | |  | | 0.446 | 0.006 | 1.23 |
|  | intra-scanner-F | 0.461 | 0.007 | 1.62 | |  | | 0.458 | 0.013 | 2.91 |
|  | intra-scanner-G | 0.479 | 0.011 | 2.37 | |  | | 0.474 | 0.014 | 2.92 |
| posterior limb internal capsule | inter-scanner | 0.577 | 0.016 | 2.85 | |  | | 0.585 | 0.029 | 5.02 |
|  | intra-scanner-A | 0.568 | 0.011 | 1.98 | |  | | 0.543 | 0.014 | 2.61 |
|  | intra-scanner-C | 0.578 | 0.026 | 4.46 | |  | | 0.546 | 0.008 | 1.39 |
|  | intra-scanner-D | 0.558 | 0.006 | 1.10 | |  | | 0.587 | 0.007 | 1.13 |
|  | intra-scanner-E | 0.582 | 0.007 | 1.20 | |  | | 0.601 | 0.002 | 0.32 |
|  | intra-scanner-F | 0.577 | 0.017 | 2.89 | |  | | 0.597 | 0.025 | 4.17 |
|  | intra-scanner-G | 0.597 | 0.009 | 1.47 | |  | | 0.610 | 0.012 | 2.02 |
| sagittal stratum | inter-scanner | 0.497 | 0.026 | 5.31 | |  | | 0.459 | 0.021 | 4.53 |
|  | intra-scanner-A | 0.484 | 0.007 | 1.46 | |  | | 0.435 | 0.009 | 2.09 |
|  | intra-scanner-C | 0.518 | 0.003 | 0.56 | |  | | 0.476 | 0.010 | 2.19 |
|  | intra-scanner-D | 0.492 | 0.014 | 2.83 | |  | | 0.459 | 0.010 | 2.18 |
|  | intra-scanner-E | 0.463 | 0.044 | 9.45 | |  | | 0.449 | 0.020 | 4.55 |
|  | intra-scanner-F | 0.507 | 0.008 | 1.50 | |  | | 0.462 | 0.019 | 4.13 |
|  | intra-scanner-G | 0.522 | 0.004 | 0.82 | |  | | 0.479 | 0.018 | 3.78 |
| uncinate fasciculus | inter-scanner | 0.413 | 0.023 | 5.67 | |  | | 0.383 | 0.024 | 6.23 |
|  | intra-scanner-A | 0.407 | 0.013 | 3.10 | |  | | 0.364 | 0.009 | 2.50 |
|  | intra-scanner-C | 0.426 | 0.007 | 1.76 | |  | | 0.377 | 0.009 | 2.43 |
|  | intra-scanner-D | 0.405 | 0.002 | 0.50 | |  | | 0.377 | 0.005 | 1.22 |
|  | intra-scanner-E | 0.414 | 0.043 | 10.28 | |  | | 0.399 | 0.024 | 6.05 |
|  | intra-scanner-F | 0.403 | 0.018 | 4.41 | |  | | 0.373 | 0.021 | 5.66 |
|  | intra-scanner-G | 0.438 | 0.016 | 3.67 | |  | | 0.411 | 0.023 | 5.65 |
| corpus callosum | inter-scanner | 0.605 | 0.019 | 3.11 | |  | |  |  |  |
|  | intra-scanner-A | 0.569 | 0.006 | 1.06 | |  | |  |  |  |
|  | intra-scanner-C | 0.597 | 0.011 | 1.91 | |  | |  |  |  |
|  | intra-scanner-D | 0.612 | 0.011 | 1.74 | |  | |  |  |  |
|  | intra-scanner-E | 0.618 | 0.005 | 0.84 | |  | |  |  |  |
|  | intra-scanner-F | 0.607 | 0.008 | 1.28 | |  | |  |  |  |
|  | intra-scanner-G | 0.620 | 0.005 | 0.77 | |  | |  |  |  |

CV=coefficient of variation, FA=fractional anisotropy.

Supplemental Table 3. Inter and intra-scanner variability of MD in white matter ROIs. All scans were included (n=24). DTI data were not available Scanner B. MD is scaled X 1000 mm^2^/s.

|  |  | LEFT | | |  | | RIGHT | | | |
| --- | --- | --- | --- | --- | --- | --- | --- | --- | --- | --- |
|  |  | Mean MD | SD MD | CV(%) | |  | | Mean MD | SD MD | CV(%) |
| anterior limb internal capsule | inter-scanner | 0.725 | 0.061 | 8.43 | |  | | 0.736 | 0.055 | 7.42 |
|  | intra-scanner-A | 0.632 | 0.009 | 1.49 | |  | | 0.662 | 0.011 | 1.68 |
|  | intra-scanner-C | 0.695 | 0.022 | 3.24 | |  | | 0.694 | 0.028 | 4.00 |
|  | intra-scanner-D | 0.739 | 0.007 | 1.01 | |  | | 0.768 | 0.012 | 1.58 |
|  | intra-scanner-E | 0.701 | 0.016 | 2.34 | |  | | 0.697 | 0.006 | 0.92 |
|  | intra-scanner-F | 0.736 | 0.014 | 1.95 | |  | | 0.748 | 0.012 | 1.56 |
|  | intra-scanner-G | 0.828 | 0.013 | 1.53 | |  | | 0.822 | 0.015 | 1.85 |
| arcuate fasciculus region 1 | inter-scanner | 0.715 | 0.044 | 6.17 | |  | | 0.733 | 0.035 | 4.73 |
|  | intra-scanner-A | 0.661 | 0.010 | 1.44 | |  | | 0.677 | 0.004 | 0.57 |
|  | intra-scanner-C | 0.696 | 0.002 | 0.24 | |  | | 0.733 | 0.002 | 0.21 |
|  | intra-scanner-D | 0.767 | 0.005 | 0.65 | |  | | 0.777 | 0.005 | 0.61 |
|  | intra-scanner-E | 0.680 | 0.003 | 0.43 | |  | | 0.711 | 0.008 | 1.15 |
|  | intra-scanner-F | 0.714 | 0.016 | 2.20 | |  | | 0.737 | 0.012 | 1.63 |
|  | intra-scanner-G | 0.779 | 0.015 | 1.95 | |  | | 0.771 | 0.009 | 1.15 |
| arcuate fasciculus region 2 | inter-scanner | 0.747 | 0.030 | 3.99 | |  | | 0.733 | 0.024 | 3.34 |
|  | intra-scanner-A | 0.699 | 0.012 | 1.72 | |  | | 0.689 | 0.007 | 1.07 |
|  | intra-scanner-C | 0.735 | 0.012 | 1.68 | |  | | 0.747 | 0.020 | 2.61 |
|  | intra-scanner-D | 0.776 | 0.010 | 1.27 | |  | | 0.751 | 0.001 | 0.20 |
|  | intra-scanner-E | 0.747 | 0.014 | 1.81 | |  | | 0.722 | 0.015 | 2.02 |
|  | intra-scanner-F | 0.744 | 0.010 | 1.32 | |  | | 0.743 | 0.008 | 1.06 |
|  | intra-scanner-G | 0.787 | 0.009 | 1.16 | |  | | 0.750 | 0.014 | 1.87 |
| arcuate fasciculus region 3 | inter-scanner | 0.753 | 0.032 | 4.30 | |  | | 0.759 | 0.027 | 3.55 |
|  | intra-scanner-A | 0.694 | 0.006 | 0.84 | |  | | 0.706 | 0.006 | 0.78 |
|  | intra-scanner-C | 0.740 | 0.002 | 0.33 | |  | | 0.768 | 0.000 | 0.04 |
|  | intra-scanner-D | 0.768 | 0.014 | 1.77 | |  | | 0.764 | 0.006 | 0.78 |
|  | intra-scanner-E | 0.764 | 0.027 | 3.53 | |  | | 0.753 | 0.006 | 0.81 |
|  | intra-scanner-F | 0.757 | 0.012 | 1.57 | |  | | 0.772 | 0.014 | 1.79 |
|  | intra-scanner-G | 0.787 | 0.012 | 1.50 | |  | | 0.783 | 0.010 | 1.23 |
| cingulum | inter-scanner | 0.762 | 0.039 | 5.18 | |  | | 0.746 | 0.043 | 5.82 |
|  | intra-scanner-A | 0.689 | 0.006 | 0.82 | |  | | 0.668 | 0.005 | 0.79 |
|  | intra-scanner-C | 0.751 | 0.010 | 1.29 | |  | | 0.727 | 0.004 | 0.50 |
|  | intra-scanner-D | 0.781 | 0.010 | 1.33 | |  | | 0.771 | 0.007 | 0.86 |
|  | intra-scanner-E | 0.771 | 0.007 | 0.96 | |  | | 0.749 | 0.007 | 0.96 |
|  | intra-scanner-F | 0.763 | 0.015 | 1.98 | |  | | 0.748 | 0.017 | 2.25 |
|  | intra-scanner-G | 0.813 | 0.014 | 1.78 | |  | | 0.805 | 0.009 | 1.14 |
| posterior limb internal capsule | inter-scanner | 0.719 | 0.048 | 6.71 | |  | | 0.703 | 0.039 | 5.53 |
|  | intra-scanner-A | 0.626 | 0.006 | 0.95 | |  | | 0.639 | 0.011 | 1.75 |
|  | intra-scanner-C | 0.698 | 0.006 | 0.87 | |  | | 0.717 | 0.009 | 1.23 |
|  | intra-scanner-D | 0.763 | 0.013 | 1.68 | |  | | 0.709 | 0.010 | 1.46 |
|  | intra-scanner-E | 0.717 | 0.008 | 1.15 | |  | | 0.679 | 0.002 | 0.26 |
|  | intra-scanner-F | 0.731 | 0.009 | 1.23 | |  | | 0.713 | 0.009 | 1.23 |
|  | intra-scanner-G | 0.768 | 0.009 | 1.16 | |  | | 0.761 | 0.012 | 1.61 |
| sagittal stratum | inter-scanner | 0.834 | 0.050 | 6.01 | |  | | 0.811 | 0.040 | 4.96 |
|  | intra-scanner-A | 0.739 | 0.015 | 2.07 | |  | | 0.728 | 0.006 | 0.76 |
|  | intra-scanner-C | 0.796 | 0.017 | 2.17 | |  | | 0.817 | 0.015 | 1.80 |
|  | intra-scanner-D | 0.877 | 0.021 | 2.37 | |  | | 0.819 | 0.013 | 1.58 |
|  | intra-scanner-E | 0.837 | 0.010 | 1.23 | |  | | 0.827 | 0.006 | 0.75 |
|  | intra-scanner-F | 0.859 | 0.017 | 2.02 | |  | | 0.831 | 0.011 | 1.34 |
|  | intra-scanner-G | 0.870 | 0.008 | 0.97 | |  | | 0.833 | 0.027 | 3.22 |
| uncinate fasciculus | inter-scanner | 0.799 | 0.042 | 5.21 | |  | | 0.811 | 0.032 | 3.91 |
|  | intra-scanner-A | 0.735 | 0.008 | 1.04 | |  | | 0.778 | 0.008 | 1.02 |
|  | intra-scanner-C | 0.790 | 0.007 | 0.88 | |  | | 0.794 | 0.026 | 3.29 |
|  | intra-scanner-D | 0.810 | 0.008 | 1.01 | |  | | 0.821 | 0.015 | 1.83 |
|  | intra-scanner-E | 0.778 | 0.050 | 6.46 | |  | | 0.786 | 0.035 | 4.51 |
|  | intra-scanner-F | 0.817 | 0.012 | 1.44 | |  | | 0.819 | 0.012 | 1.46 |
|  | intra-scanner-G | 0.901 | 0.059 | 6.51 | |  | | 0.857 | 0.011 | 1.28 |
| corpus callosum | inter-scanner | 0.789 | 0.017 | 2.15 | |  | |  |  |  |
|  | intra-scanner-A | 0.877 | 0.008 | 0.92 | |  | |  |  |  |
|  | intra-scanner-C | 0.917 | 0.013 | 1.44 | |  | |  |  |  |
|  | intra-scanner-D | 0.925 | 0.006 | 0.66 | |  | |  |  |  |
|  | intra-scanner-E | 0.910 | 0.020 | 2.21 | |  | |  |  |  |
|  | intra-scanner-F | 0.972 | 0.009 | 0.90 | |  | |  |  |  |
|  | intra-scanner-G | 0.725 | 0.061 | 8.43 | |  | |  |  |  |

CV=coefficient of variation, MD=mean diffusivity (mm^2^/s).
